# Feasibility and safety of in-bed cycling/stepping in critically ill patients: A study protocol for a pilot randomized controlled clinical trial

**DOI:** 10.1371/journal.pone.0301368

**Published:** 2024-05-10

**Authors:** Soohyun Wi, Hyung-Ik Shin, Sung Eun Hyun, Kwan-Sik Sung, Woo Hyung Lee

**Affiliations:** 1 Biomedical Research Institute, Seoul National University Hospital, Seoul, South Korea; 2 Department of Rehabilitation Medicine, Seoul National University College of Medicine, Seoul National University Hospital, Seoul, South Korea; University Hospital Cologne: Uniklinik Koln, GERMANY

## Abstract

**Background:**

Intensive care unit (ICU)-acquired weakness (ICU-AW) is one of the most common complications of post-ICU syndrome. It is the leading cause of gait disturbance, decreased activities of daily living, and poor health-related quality of life. The early rehabilitation of critically ill patients can reduce the ICU-AW. We designed a protocol to investigate the feasibility and safety of conventional rehabilitation with additional in-bed cycling/stepping in critically ill patients.

**Methods:**

The study is designed as a single-center, single-blind, pilot, randomized, parallel-group study. After the screening, participants are randomly allocated to two groups, stratified by mechanical ventilation status. The intervention group will be provided with exercises of in-bed cycling/stepping according to the level of consciousness, motor power, and function in addition to conventional rehabilitation. In contrast, the control group will be provided with only conventional rehabilitation. The length of intervention is from ICU admission to discharge, and interventions will be conducted for 20 minutes, a maximum of three sessions per day.

**Results:**

The outcomes are the number and percentage of completed in-bed cycling/stepping sessions, the duration and percentage of in-bed cycling/stepping sessions, and the number of cessations of in-bed cycling/stepping sessions, the interval from ICU admission to the first session of in-bed cycling/stepping, the number and percentage of completed conventional rehabilitation sessions, the duration and percentage of conventional rehabilitation sessions, the number of cessations of conventional rehabilitation sessions, the number of adverse events, level of consciousness, functional mobility, muscle strength, activities of daily living, and quality of life.

**Discussion:**

This study is a pilot clinical trial to investigate the feasibility and safety of conventional rehabilitation with additional in-bed cycling/stepping in critically ill patients. If the expected results are achieved in this study, the methods of ICU rehabilitation will be enriched.

**Trial registration:**

clinicialtrials.gov, Clinical Trials Registration #NCT05868070.

## Introduction

The prevention of post-intensive care syndrome has been gaining great interest with the improvement in survival rates of critically ill patients [1]. Post-intensive care syndrome indicates physical, cognitive, and mental complications, including critical illness neuropathy and myopathy, disuse atrophy, memory, and executive dysfunction, depression, post-traumatic stress disorder, and anxiety [2]. Intensive care unit (ICU)-acquired weakness (ICU-AW) is one of the most common complications of post-ICU syndrome [3]. It is the main cause of gait disturbance, decreased activities of daily living (ADL), and poor health-related quality of life, which may occur and even persist after ICU discharge [4].

Early rehabilitation is crucial to mitigate ICU-AW [5]. The incidence and severity of ICU-AW, along with the duration of mechanical ventilation, ICU length of stay, functional independence, and mortality, can be improved as a result of early rehabilitation [6, 7]. However, effective rehabilitation is frequently hampered in the ICU setting due to disease status, disorganized behavior, sedation, insertion of tubes and catheters in critically ill patients, and insufficient rehabilitation resources [8]. Thus, developing rehabilitation protocols and settings as a patient-tailored approach is necessary for early rehabilitation with proper dose and intensity.

As an adjunctive method to accomplish effective ICU rehabilitation, the utilization of in-bed exercise equipment has been attempted in critically ill patients. An in-bed cycle ergometer is one of the options that can be applied in the ICU [9]. It has been generally recognized to prevent contracture, increase blood circulation, improve cardiopulmonary function, and strengthen the lower limb muscles with fewer safety issues [10, 11]. Additionally, it is likely that stepping has additive effects on cycling [12], regarding the opportunity for gait imitation to perform reciprocal limb movements and mechanical stress on bones and muscles to bear body weight [13]. If both the cycle ergometer and stepper are applied together to critically ill patients, their effect on functional mobility or strength would be more enhanced than if only in-bed cycle ergometers were used.

Caution should be taken to provide early rehabilitation in the ICU setting because critically ill patients can be vulnerable to hemodynamic and respiratory changes, and endotracheal or nasogastric tubes and vascular or urinary catheters can be accidentally removed despite their low incidence [14]. The establishment of safety criteria considering vital signs, level of consciousness and cooperation, sedation state, administration of vasopressors, types of catheters or tubes in situ, and wounds can be made mandatory to determine whether to delay or terminate rehabilitation sessions [15, 16]. The safety criteria for the application of the in-bed cycle ergometer and stepper may need to be modified in consideration of the increased and frequent range of motion at the lower extremities compared to early active mobilization during exercises.

While previous clinical trials have incorporated in-bed cycling as ICU rehabilitation in critically ill patients [17, 18], few studies have been published that simultaneously employ both in-bed cycling and stepping exercises. Therefore, we designed a pilot randomized controlled clinical trial protocol to investigate the feasibility and safety of conventional rehabilitation with additional in-bed cycling/stepping in critically ill patients.

## Methods

### Trial design and study setting

This is a prospective, single-blind, assessor-blinded, add-on, single-center, two-arm parallel, pilot, randomized controlled trial. This protocol adhered to the Standard Protocol Items: Recommendations for Interventional Trials (SPIRIT) guidelines [19] (see S1 Table) and TIDier checklist [20] (see S2 Table). Inclusion criteria are critically ill patients who are aged 45 years or older, are admitted to ICU less than 72 hours before randomization, are deemed to need ICU care for more than 48 hours, and have Functional Ambulation Category (FAC) 2 or higher before admission to the ICU. Exclusion criteria are as follows: any neurological disorders, including acute stroke, advanced dementia, hypoxic-ischemic encephalopathy, amyotrophic lateral sclerosis, myasthenia gravis, and/or acute inflammatory demyelinating polyradiculoneuropathy; the presence of acute deep venous thrombosis and/or pulmonary embolism; pneumothorax; an external fixator, superficial metallic implants, amputations, and/or eschar in the lower extremities; expected ICU discharge within 3 days of admission; pregnancy; difficulty in obtaining informed consent; the life expectancy of less than six months. The inclusion and exclusion criteria are presented in Table 1. A member of the study team will obtain written consent during ICU admission for a baseline evaluation, and the research process will not proceed until consent is obtained. Through screening, the members of the research team will verify their eligibility.

**Table 1:** Inclusion and exclusion criteria.

Inclusion criteria	Exclusion criteria
• Age ≥ 45 •Admission of ICU ≤ 72 hours •Patient deemed to need ≥ 48 hours of ICU care •Premorbid Functional Ambulation Category ≥ 2	ㆍNeurological disorders ㆍ Central nervous system: acute stroke, advanced dementia, hypoxic-ischemic encephalopathy ㆍ Peripheral nervous system: amyotrophic lateral sclerosis, myasthenia gravis, acute inflammatory demyelinating polyradiculoneuropathy ㆍAcute deep venous thrombosis, pulmonary embolism ㆍPneumothorax ㆍExternal fixator, superficial metallic implants, amputation, eschar, etc. ㆍExpected ICU discharge within 3 days of admission ㆍPregnancy ㆍDifficulty in obtaining informed consent (rejection, no family, if the family does not agree) ㆍIf the life expectancy is less than 6 months

### Recruitment

Clinicians in the ICU at our hospital will collaborate to enroll patients for this clinical trial. Additionally, posters about this study are planned to be affixed on the noticeboard in front of the ICU, facilitating easy access to information regarding the clinical trial for representatives of patients. The principal investigator will provide a detailed explanation of the study to the participants’ legal representatives since most admitted patients are critically or severely ill. The study will not exclude patients who are likely to participate based on their socioeconomic status. This study is a pilot clinical trial to investigate the feasibility and safety of in-bed cycling/stepping exercise intervention and aims to recruit at least 24 participants. Assuming a 20% loss for death and a 15% loss for follow-up, the required sample size was 12 per arm to be recruited in this study.

### Allocation

The computer-generated sequence will be generated on the website (https://mrcc.snuh.org) by an independent institution in our hospital. A computer-generated sequence will randomly allocate the two groups in this study. The stratified block randomization method will assign the randomization table to the control or intervention group at a 1:1 ratio. The use of mechanical ventilation will stratify the participants.

The group allocation will be performed by an independent researcher who is not involved in evaluating outcomes and will be concealed to physiotherapists who are engaged in assessing outcomes. The participants and in-bed cycling/stepping intervention provider will be instructed not to disclose the allocation to the evaluator. After the end of the clinical trial, blinding will be maintained until the input of evaluation data is completed. Any unblinding prior to clinical trials due to accidental or serious adverse events should be documented. To promote data quality for assessment and collection of outcomes, assessors will be well-trained.

### Interventions

#### Conventional rehabilitation

**Fig 1:**
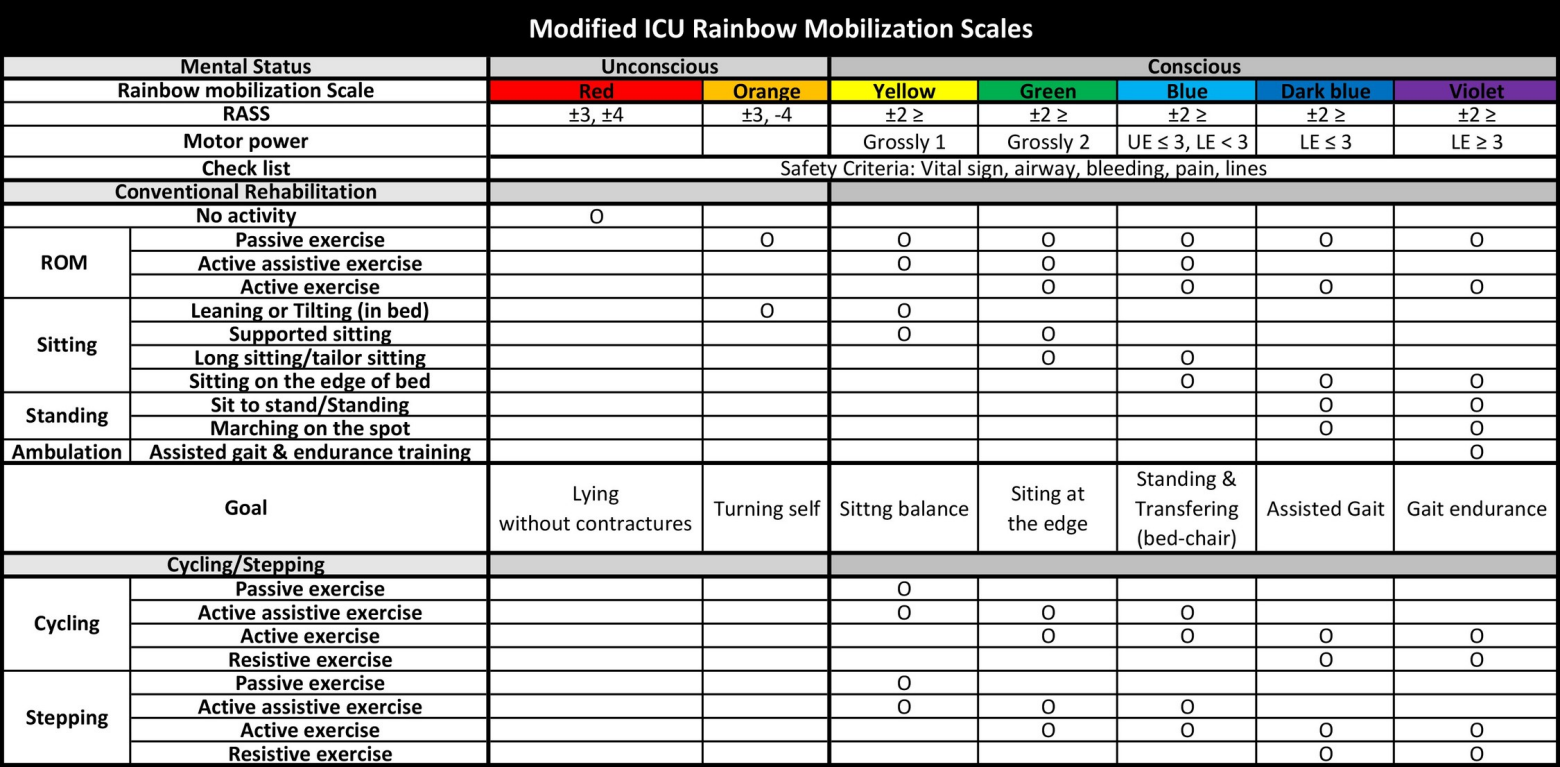
ICU rehabilitation protocol with exercise intervention using in-bed cycle ergometer/stepper. Critically ill patients will be classified from red to violet according to Richmond agitation-sedation scale (RASS), motor power, and level of consciousness. The patients in the control group will receive conventional rehabilitation according to the ICU rainbow mobilization scale program including range of motion (ROM), sitting, standing, and ambulation. Critically ill patients in the intervention group will receive in-bed cycling/stepping intervention starting from Yellow to Violet in addition to the conventional rehabilitation. As the condition of the critically ill patient improves from Yellow to Violet, the exercise intervention gradually progresses to passive, active assistive, active, and resistive exercise modes. In-bed cycling/stepping is provided by physiotherapists from ICU admission to discharge, and the routine frequency of in-bed cycling/stepping is 1–3 sessions per day on the weekdays during ICU hospitalization. The number of in-bed cycling/stepping will be increased gradually. Cycling is performed if there is one session per day; cycling-stepping is performed if there are two sessions per day; and cycling-stepping-cycling is performed if there are three sessions per day. ROM: range of motion; UE: upper extremity; LE: lower extremity.

Patients in the control group will only receive conventional rehabilitation in the ICU. The protocol for conventional rehabilitation in the ICU is shown in Fig 1. An standardized stepwise ICU rehabilitation program is provided for 20 min based on the stage of the patient’s condition, which is evaluated prior to each treatment session and considers consciousness, motor power, and functional mobility according to previous studies [10, 15]. The stage of the patient’s condition is graded as the 7-Likert scale from red (least able) to violet (most able) [21, 22], which means that the patient’s state of consciousness, muscle strength, and function improve sequentially. Generally, rehabilitation for critically ill patients is performed depending on the condition of critically ill patients [23, 24]. The goal of the conventional rehabilitation at each stage is lying without contractures, turning self, sitting balance, sitting at the edge, standing and transferring from bed to chair, assisted gait, and gait endurance. The conventional rehabilitation is provided by physiotherapists from ICU admission to hospital discharge, and the routine frequency of conventional rehabilitation is one session per day during weekdays.

#### In-bed cycling/stepping

Patients in the intervention group will receive conventional rehabilitation plus exercise intervention with an in-bed cycle ergometer/stepper for additional 20 min in the ICU. The protocol, including exercise intervention, was developed by modifying the rainbow mobilization scales and the existing intensive care rehabilitation protocol shown in Fig 1. As the condition of the critically ill patient improves from yellow to violet, the intervention gradually progresses to passive, active assistive, active, and resistive exercise. In-bed cycling/stepping is provided by physiotherapists from ICU admission to discharge, and the routine frequency of in-bed cycling/stepping is 1–3 sessions per day on the weekdays during ICU hospitalization. The number of in-bed cycling/stepping interventions will gradually be increased depending on the patient’s condition [23]. Cycling is performed if there is one session per day; cycling-stepping if there are two sessions per day; and cycling-stepping-cycling if there are three sessions per day. During the intervention, the target rating of perceived exertion ranges from moderate to hard, and the exercise intensity is adjusted in this range [25, 26].

Risk criteria for delaying or terminating ICU rehabilitation will be assessed to determine patients’ suitability to participate in rehabilitation before and during rehabilitation sessions (Table 2) [15, 16]. ICU rehabilitation should be delayed or ceased if there is no recovery within two minutes under any of the conditions. Adverse events will be recorded after rehabilitation sessions. To improve intervention adherence, the participants are provided with an exercise log. Usual care, such as pulmonary rehabilitation and occupational therapy, will be maintained for all patients during the trial. Additionally, usual care will be maintained for all patients following trial completion.

**Table 2:** Criteria for delaying or terminating ICU rehabilitation and exercise intervention.

Respiratory conditions
ㆍPercutaneous oxygen saturation (SpO_2_) < 88% ㆍRespiratory rate (RR) > 40bpm ㆍUnsecure airway
**Cardiovascular conditions**
ㆍMean arterial pressure (MAP) < 60mmHg or > 120mmHg ㆍSystolic blood pressure (SBP) < 90mmHg or > 200mmHg ㆍHeart rate (HR) < 50bpm or > 140bpm ㆍArrhythmias causing hemodynamic instability ㆍConcerns about new myocardial ischemia
**Neurologic conditions**
ㆍ Richmond agitation-sedation scale (RASS) scale ≥ 3 ㆍUncontrolled epilepsy
**Others**
ㆍActive bleeding, such as Gastrointestinal bleeding or Overt bleeding ㆍBody temperature such as ≥ 39° ㆍNeuromuscular blockage within 4 hours ㆍPain, severe nausea and vomiting ㆍVentilator asynchrony or risk of extubation or weaning of ventilator ㆍOpen wounds on the lower extremities ㆍFall ㆍRequest to stop exercising
**Catheter placement in the femoral artery or vein (for only exercise intervention)**
ㆍExtracorporeal membrane oxygenation of the femoral artery or the presence of an intra-aortic balloon pump ㆍPerm catheter inserted into the femoral vein

### Outcome measures

Baseline data will be measured prior to randomization, and follow-up data will be collected at ICU discharge, hospital discharge, and 1 and 3 months after hospital discharge. The following baseline clinical characteristics will be collected, shown in Table 3 [7, 23]: age, sex, weight, height, premorbid modified Barthel index, premorbid FAC, Acute Physiology and Chronic Health Evaluation (APACHE II) score [27], whether cardiopulmonary resuscitation (CPR) was performed within 24 hours before admission to the ICU, primary diagnostic category of ICU admission, and comorbidity, rehabilitation services after hospital discharge.

**Table 3:** Clinical examination items.

**Baseline clinical characteristics**
ㆍBasic information: age, sex, weight, heightㆍPremorbid modified Barthel index (MBI)ㆍPremorbid functional ambulation category (FAC)ㆍ The Acute Physiology and Chronic Health Evaluation (APACHE II) scoreㆍCardio-pulmonary resuscitation (CPR) within 24 hours prior to admission to the ICU •Primary diagnostic category of ICU Admission •Comorbidity •Geriatric disease
**Outcomes**
ㆍThe number and percentage of completed in-bed cycling/stepping sessions ㆍThe duration and percentage of in-bed cycling/stepping sessions ㆍThe number of cessations of in-bed cycling/stepping sessions
ㆍThe interval from ICU admission to the first session of in-bed cycling/stepping ㆍ The number and percentage of completed conventional rehabilitation sessions ㆍ The duration and percentage of conventional rehabilitation sessions ㆍ The number of cessations of conventional rehabilitation sessions ㆍ The number of adverse events ㆍ Level of consciousness: Richmond agitation-sedation scale (RASS), confusion assessment method for the ICU (CAM-ICU) ㆍ Mobility: functional ambulatory category (FAC), functional status score (FSS-ICU), short physical performance battery score (SPPB), de Morton Mobility Index (DEMMI), falls efficacy scale (FES), activities-specific balance confidence scale (ABC) ㆍMotor power: sum of Medical Research Council (MRC) score, hand grip strength (HGS) ㆍActivities of daily living (ADL): modified Barthel index (MBI) ㆍQuality of life (QOL): 36-item short form survey (SF-36) version 2.0 ㆍOthers: Pittsburgh rehabilitation participation scale (PRPS), days to initiate ambulation (FAC ≥2), mortality-28 days, duration of mechanical ventilation, length of stay in the ICU, length of stay in the hospital, concomitant occupational therapy and its application dose, concomitant pulmonary rehabilitation and its application dose

The outcomes listed in Table 3 are the number and percentage of completed in-bed cycling/stepping sessions, the duration and percentage of in-bed cycling/stepping sessions, and the number of cessations of in-bed cycling/stepping sessions, the interval from ICU admission to the first session of in-bed cycling/stepping, the number and percentage of completed conventional rehabilitation sessions, the duration and percentage of conventional rehabilitation sessions, the number of cessations of conventional rehabilitation sessions, the number of adverse events, the confusion assessment method for the ICU (CAM-ICU), Richmond agitation-sedation scale (RASS), de Morton mobility index (DEMMI), FAC, functional status score for the ICU (FSS-ICU), short physical performance battery score (SPPB), falls efficacy scale (FES), activities-specific balance confidence scale (ABC), sum of Medical Research Council (MRC) score, handgrip strength, modified Barthel index, 36-item short-form survey (SF-36) version 2.0, days to initiate ambulation (FAC ≥2), mortality-28 days, duration of mechanical ventilation, length of stay in the ICU, length of stay in the hospital, Pittsburgh rehabilitation participation scale (PRPS), concomitant occupational therapy and its application dose, concomitant pulmonary rehabilitation and its application dose.

**Fig 2:**
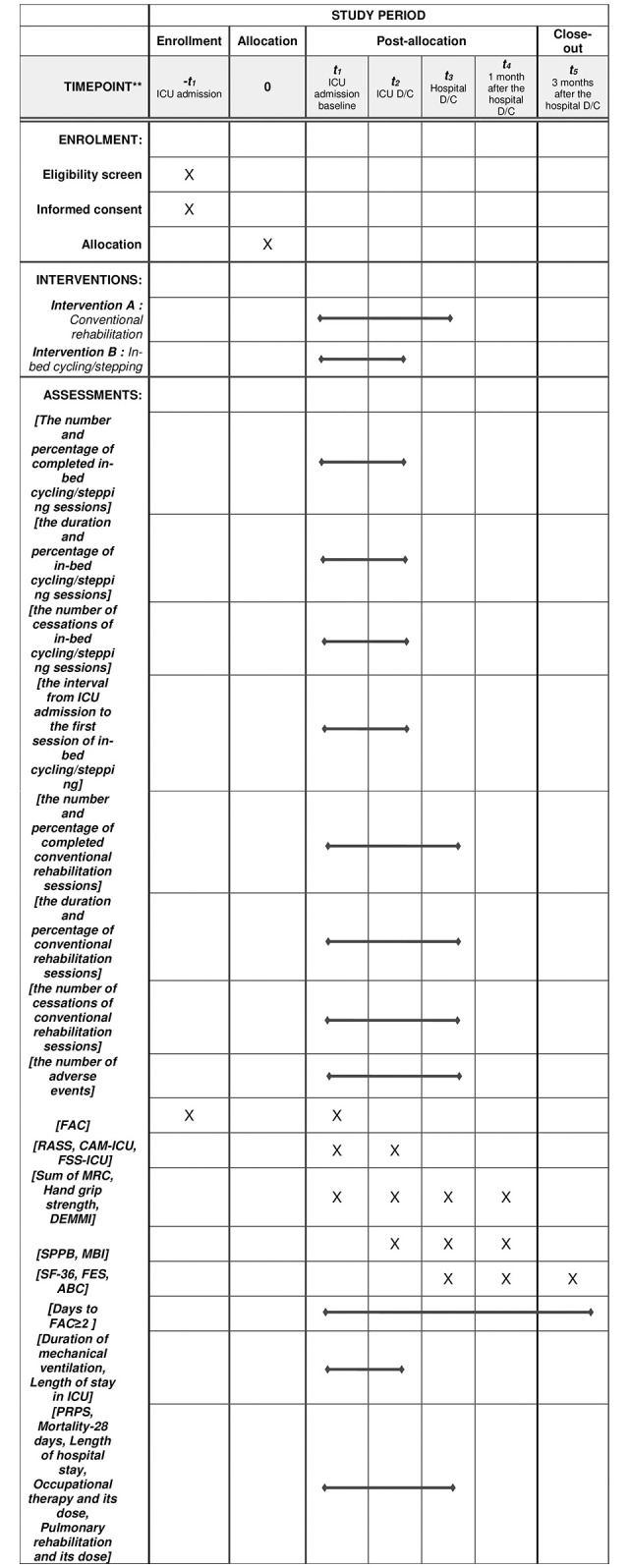
SPIRIT schedule of enrolment, interventions, and assessments of the study. The outcomes are the number and percentage of completed in-bed cycling/stepping sessions, the duration and percentage of in-bed cycling/stepping sessions, and the number of cessations of in-bed cycling/stepping sessions, the interval from ICU admission to the first session of in-bed cycling/stepping, the number and percentage of completed conventional rehabilitation sessions, the duration and percentage of conventional rehabilitation sessions, the number of cessations of conventional rehabilitation sessions, the number of adverse events, CAM-ICU, RASS, FAC, FSS-ICU, DEMMI, FES, ABC, Sum of MRC, MBI, SF-36 version 2.0, PRPS, days to initiate ambulation (FAC≥2) and others. ICU: intensive care unit; D/C: discharge; FAC: functional ambulation category; RASS: Richmond agitation-sedation scale; CAM-ICU: confusion assessment method for the ICU; MRC: Medical Research Council; DEMMI: de Morton mobility index; FSS-ICU: functnal status score for the ICU; SPPB: short physical performance battery score; MBI: modified Barthel index; SF-36: 36-item short form survey; FES: falls efficacy scale; ABC: activities-specific balance confidence scale; PRPS: Pittsburgh rehabilitation participation scale.

The schedule of enrollment and assessment of the participants are shown in Fig 2. After ICU admission, patients are screened according to the inclusion criteria. After the screening, the guardians of patients who meet the inclusion criteria will be given an information sheet and asked to provide written consent to participate in the trial. Patients will be enrolled in the trial, given a trial-specific identification number, and randomly allocated to a group using a computer-generated random sequence that is listed prior to the start of the trial. After baseline evaluation, patients will be assessed at ICU discharge, hospital discharge, 1 month after hospital discharge, and 3 months after hospital discharge. Measurements 1 month after hospital discharge will be conducted by a visit and measurements 3 months after hospital discharge will be performed over the phone.

Data are collected and recorded on a standard report form and all the recorded data will be entered into the web-based data system by the double-entry method. All errors will need to be corrected by crossing out, with the researcher’s signature and date.

### Adverse events

In this clinical trial, adverse events are all undesirable medical events that cause symptoms that were not observed before the start of the clinical trial, listed in Table 4. Predicted adverse events include falls, fractures, severe pain, endotracheal tube removal, arterial line removal, central line removal, arrhythmia, bradycardia, systolic blood pressure > 200 mmHg, systolic blood pressure < 90 mmHg due to hemodynamic instability during exercise, desaturation ≤ 88% due to respiratory instability during exercise and other minor adverse reactions, such as dyspnea, dizziness, tachypnea, and sinus tachycardia [7, 28, 29]. Predicted side effects are also classified as adverse events, and the severity of adverse reactions is classified into mild, moderate, and severe. The frequency of adverse events that are both relevant and irrelevant to the investigational device will be recorded. In the case of unexpected serious adverse events or other unintended trial interventions over the course of the study, we will report to the institutional review board. Data and safety monitoring will be conducted for every five-participant registration.

**Table 4:** Lists of predicted adverse events.

Adverse events
Fall •Fracture •Severe pain •Endotracheal tube removal •Arterial line removal •Central line removal •Arrhythmia •Bradycardia •Systolic blood pressure > 200 mmHg or < 90 mmHg due to hemodynamic instability during exercise •Desaturation ≤ 88% due to respiratory instability during exercise •Other minor adverse reactions such as dyspnea, dizziness, tachypnea, and sinus tachycardia

### Ethics approval and consent to participate

This study will be approved by the Ethics and Research Committee of the responsible institution (IRB number 2303-176-1419). The consent form for informed consent will be approved by the responsible ethics committee.

### Statistical methods

Statistical analysis in this study will be conducted using SPSS (version 25.0; IBM, Armonk, NY, USA). All statistical analyses will be performed for an intention to treat and a per protocol population. The outcomes of the continuous variables will be analyzed using Mann-Whitney U test, whereas the discontinuous variables will be analyzed using the chi-square test or Fisher’s exact test for variables between the two groups at the same time. Additionally, subgroup analysis will be conducted between the stratified groups Multiple imputation by chained equations algorithm will be used to handle missing data. The datasets analyzed and codes used for these analyses will be available from the corresponding author upon reasonable request.

## Discussion

This is the study protocol to investigate the feasibility and safety of conventional rehabilitation with additional in-bed cycling/stepping in critically ill patients. Due to the dynamic nature of critical illness, the initiation and dosage of early rehabilitation may vary. To examine the feasibility and safety of the intervention, the outcomes of this study were as follows: the number and percentage of completed in-bed cycling/stepping sessions, the duration and percentage of in-bed cycling/stepping sessions, and the number of cessations of in-bed cycling/stepping sessions. Several previous clinical studies have reported that in-bed cycling is feasible in critical illness patients [9, 17, 30–32]. Patients received in-bed cycling with approximately 5–6 sessions, completing 79–90% of planned sessions on average in the ICU [17, 31, 33]. Additionally, in-bed cycling is safe and has a low rate of ceased sessions and adverse events in critically ill patients [9, 23, 31, 33, 34]. Detailed safety criteria and guidelines for delay or cease of the intervention may contribute to the safe implementation of the in-bed cycling/stepping intervention in the protocol of this study (S1 File).

Previously, most clinical trials using in-bed equipment for rehabilitation in critically ill patients utilized the in-bed cycle ergometer; however, these studies did not reveal a significant difference in functional mobility or muscle strength between the intervention and control groups [23, 35]. One of the reasons why previous studies did not show clinically significant changes is that a single mode of physical activity in terms of in-bed cycling may not be sufficient to improve functional mobility or muscle strength in critically ill patients. Current exercise guidelines for healthy individuals recommend various elements of physical activity to enhance overall physical fitness and adherence and reduce tissue injury [36]. However, the application of various exercise programs based on multiple modes of physical activity can be highly limited in bedridden patients in ICU settings, even though early rehabilitation can be performed with the assistance of physical therapists [37]. In-bed cycling/stepping along with early rehabilitation can be a good option to enable critically ill patients to perform multiple modes of exercise. Cycling can be helpful in preventing contractures, increasing blood circulation, improving cardiorespiratory function, and strengthening lower extremity muscles, while stepping can provide reciprocal movements of the lower extremities and mechanical stress to bones and muscles by increasing body weight. Additive or synergistic effects can be expected by applying in-bed cycling/stepping together in ICU settings [38]. Further study is required to investigate the effectiveness of in-bed cycling/stepping interventions in critically ill patients and to determine the optimal dosage of passive, active, and resistive exercises tailored to the condition of the patients.

## Supporting information

S1 Table SPIRIT checklist. (DOC)

S2 Table Template for Intervention Description and Replication (TIDieR) checklist. (DOCX)

S1 File Detailed protocol submitted to the ethics committee. (PDF)

## Abbreviations

ABC: activities-specific balance confidence scale
APACHE II: Acute Physiology and Chronic Health Evaluation
CAM-ICU: confusion assessment method for the ICU
CPR: cardiopulmonary resuscitation
DEMMI: de Morton mobility index
FAC: functional ambulation category
FES: falls efficacy scale
FSS-ICU: functional status score for the ICU
HGS: hand grip strength
ICU: intensive care unit
ICU-AW: intensive care unit (ICU)-acquired weakness
MBI: modified Barthel index
MRC: Medical Research Council
SF-36: 36-item short form survey
SPPB: short physical performance battery score
PICS: post-intensive care syndrome
PRPS: Pittsburgh rehabilitation participation scale
RASS: Richmond agitation-sedation scale

## References

[1] Zimmerman JE, Kramer AA, Knaus WA. Changes in hospital mortality for United States intensive care unit admissions from 1988 to 2012. Crit Care. 2013;17(2):R81. Epub 2013/04/30. doi: 10.1186/cc12695 ; PubMed Central PMCID: PMC4057290. 23622086 PMC4057290

[2] Needham DM, Davidson J, Cohen H, Hopkins RO, Weinert C, Wunsch H, et al. Improving long-term outcomes after discharge from intensive care unit: report from a stakeholders’ conference. Crit Care Med. 2012;40(2):502–9. Epub 2011/09/29. doi: 10.1097/CCM.0b013e318232da75 . 21946660

[3] Rawal G, Yadav S, Kumar R. Post-intensive Care Syndrome: an Overview. J Transl Int Med. 2017;5(2):90–2. Epub 2017/07/20. doi: 10.1515/jtim-2016-0016 ; PubMed Central PMCID: PMC5506407. 28721340 PMC5506407

[4] Desai SV, Law TJ, Needham DM. Long-term complications of critical care. Crit Care Med. 2011;39(2):371–9. Epub 2010/10/21. doi: 10.1097/CCM.0b013e3181fd66e5 . 20959786

[5] Anekwe DE, Biswas S, Bussieres A, Spahija J. Early rehabilitation reduces the likelihood of developing intensive care unit-acquired weakness: a systematic review and meta-analysis. Physiotherapy. 2020;107:1–10. Epub 2020/03/07. doi: 10.1016/j.physio.2019.12.004 . 32135387

[6] Needham DM. Mobilizing patients in the intensive care unit: improving neuromuscular weakness and physical function. JAMA. 2008;300(14):1685–90. Epub 2008/10/09. doi: 10.1001/jama.300.14.1685 . 18840842

[7] Schweickert WD, Pohlman MC, Pohlman AS, Nigos C, Pawlik AJ, Esbrook CL, et al. Early physical and occupational therapy in mechanically ventilated, critically ill patients: a randomised controlled trial. Lancet. 2009;373(9678):1874–82. Epub 2009/05/19. doi: 10.1016/S0140-6736(09)60658-9 ; PubMed Central PMCID: PMC9906655. 19446324 PMC9906655

[8] Knott A, Stevenson M, Harlow SK. Benchmarking rehabilitation practice in the intensive care unit. J Intensive Care Soc. 2015;16(1):24–30. Epub 2015/02/01. doi: 10.1177/1751143714553901 ; PubMed Central PMCID: PMC5593287. 28979371 PMC5593287

[9] Kho ME, Martin RA, Toonstra AL, Zanni JM, Mantheiy EC, Nelliot A, et al. Feasibility and safety of in-bed cycling for physical rehabilitation in the intensive care unit. J Crit Care. 2015;30(6):1419 e1-5. Epub 20150729. doi: 10.1016/j.jcrc.2015.07.025 . 26318234

[10] Kim SC, Lee SY, Lee YI. Leg muscle activation and distance setting of the leg cycle ergometer for use by the elderly. J Phys Ther Sci. 2014;26(10):1593–5. Epub 2014/11/05. doi: 10.1589/jpts.26.1593 ; PubMed Central PMCID: PMC4210406. 25364121 PMC4210406

[11] Walsh JA, McAndrew DJ, Henness DJ, Shemmell J, Cuicuri D, Stapley PJ. A Semi-recumbent Eccentric Cycle Ergometer Instrumented to Isolate Lower Limb Muscle Contractions to the Appropriate Phase of the Pedal Cycle. Front Physiol. 2021;12:756805. Epub 2021/12/17. doi: 10.3389/fphys.2021.756805 ; PubMed Central PMCID: PMC8667581. 34912239 PMC8667581

[12] Oldenburg FA, McCormack DW, Morse JL, Jones NL. A comparison of exercise responses in stairclimbing and cycling. J Appl Physiol Respir Environ Exerc Physiol. 1979;46(3):510–6. Epub 1979/03/01. doi: 10.1152/jappl.1979.46.3.510 438020

[13] Straube DD, Holleran CL, Kinnaird CR, Leddy AL, Hennessy PW, Hornby TG. Effects of dynamic stepping training on nonlocomotor tasks in individuals poststroke. Phys Ther. 2014;94(7):921–33. Epub 2014/03/15. doi: 10.2522/ptj.20130544 . 24627428

[14] Nydahl P, Sricharoenchai T, Chandra S, Kundt FS, Huang M, Fischill M, et al. Safety of Patient Mobilization and Rehabilitation in the Intensive Care Unit. Systematic Review with Meta-Analysis. Ann Am Thorac Soc. 2017;14(5):766–77. Epub 2017/02/24. doi: 10.1513/AnnalsATS.201611-843SR . 28231030

[15] Hodgson CL, Stiller K, Needham DM, Tipping CJ, Harrold M, Baldwin CE, et al. Expert consensus and recommendations on safety criteria for active mobilization of mechanically ventilated critically ill adults. Crit Care. 2014;18(6):658. Epub 20141204. doi: 10.1186/s13054-014-0658-y ; PubMed Central PMCID: PMC4301888. 25475522 PMC4301888

[16] Yang R, Zheng Q, Zuo D, Zhang C, Gan X. Safety Assessment Criteria for Early Active Mobilization in Mechanically Ventilated ICU Subjects. Respir Care. 2021;66(2):307–15. Epub 20200908. doi: 10.4187/respcare.07888 . 32900917

[17] Nickels MR, Aitken LM, Barnett AG, Walsham J, McPhail SM. Acceptability, safety, and feasibility of in-bed cycling with critically ill patients. Aust Crit Care. 2020;33(3):236–43. Epub 20200418. doi: 10.1016/j.aucc.2020.02.007 . 32317212

[18] Kimawi I, Lamberjack B, Nelliot A, Toonstra AL, Zanni J, Huang M, et al. Safety and Feasibility of a Protocolized Approach to In-Bed Cycling Exercise in the Intensive Care Unit: Quality Improvement Project. Phys Ther. 2017;97(6):593–602. doi: 10.1093/ptj/pzx034 . 28379571

[19] Chan AW, Tetzlaff JM, Gotzsche PC, Altman DG, Mann H, Berlin JA, et al. SPIRIT 2013 explanation and elaboration: guidance for protocols of clinical trials. BMJ. 2013;346:e7586. Epub 2013/01/11. doi: 10.1136/bmj.e7586 ; PubMed Central PMCID: PMC3541470. 23303884 PMC3541470

[20] Hoffmann TC, Glasziou PP, Boutron I, Milne R, Perera R, Moher D, et al. Better reporting of interventions: template for intervention description and replication (TIDieR) checklist and guide. BMJ. 2014; 348:g1687. doi: 10.1136/bmj.g1687 24609605

[21] Hiser S, Chung CR, Toonstra A, Friedman LA, Colantuoni E, Hoyer E, et al. Inter-rater reliability of the Johns Hopkins Highest Level of Mobility Scale (JH-HLM) in the intensive care unit. Braz J Phys Ther. 2021;25(3):352–5. Epub 2020/08/20. doi: 10.1016/j.bjpt.2020.07.010 ; PubMed Central PMCID: PMC8134787. 32811787 PMC8134787

[22] Hiser S, Toonstra A, Friedman LA, Colantuoni E, Needham DM. Inter-rater reliability of activity measure for post-acute care ’6-Clicks’ inpatient mobility short form in the intensive care unit. Physiother Res Int. 2020;25(4):e1849. Epub 2020/05/26. doi: 10.1002/pri.1849 ; PubMed Central PMCID: PMC9115971. 32449231 PMC9115971

[23] Fossat G, Baudin F, Courtes L, Bobet S, Dupont A, Bretagnol A, et al. Effect of In-Bed Leg Cycling and Electrical Stimulation of the Quadriceps on Global Muscle Strength in Critically Ill Adults: A Randomized Clinical Trial. JAMA. 2018;320(4):368–78. doi: 10.1001/jama.2018.9592 ; PubMed Central PMCID: PMC6583091. 30043066 PMC6583091

[24] Miranda Rocha AR, Martinez BP, Maldaner da Silva VZ, Forgiarini Junior LA. Early mobilization: Why, what for and how? Med Intensiva. 2017;41(7):429–36. Epub 2017/03/12. doi: 10.1016/j.medin.2016.10.003 . 28283324

[25] Berney S, Haines K, Skinner EH, Denehy L. Safety and feasibility of an exercise prescription approach to rehabilitation across the continuum of care for survivors of critical illness. Phys Ther. 2012;92(12):1524–35. Epub 2012/08/11. doi: 10.2522/ptj.20110406 . 22879441

[26] Nes BM, Janszky I, Wisloff U, Stoylen A, Karlsen T. Age-predicted maximal heart rate in healthy subjects: The HUNT fitness study. Scand J Med Sci Sports. 2013;23(6):697–704. Epub 2012/03/02. doi: 10.1111/j.1600-0838.2012.01445.x . 22376273

[27] Knaus WA, Draper EA, Wagner DP, Zimmerman JE. APACHE II: a severity of disease classification system. Crit Care Med. 1985;13(10):818–29. Epub 1985/10/01. . 3928249

[28] Schaller SJ, Anstey M, Blobner M, Edrich T, Grabitz SD, Gradwohl-Matis I, et al. Early, goal-directed mobilisation in the surgical intensive care unit: a randomised controlled trial. Lancet. 2016;388(10052):1377–88. Epub 2016/10/07. doi: 10.1016/S0140-6736(16)31637-3 . 27707496

[29] Bailey P, Thomsen GE, Spuhler VJ, Blair R, Jewkes J, Bezdjian L, et al. Early activity is feasible and safe in respiratory failure patients. Crit Care Med. 2007;35(1):139–45. Epub 2006/11/30. doi: 10.1097/01.CCM.0000251130.69568.87 . 17133183

[30] Ringdal M, Warren Stomberg M, Egnell K, Wennberg E, Zatterman R, Rylander C. In-bed cycling in the ICU; patient safety and recollections with motivational effects. Acta Anaesthesiol Scand. 2018;62(5):658–65. Epub 20180118. doi: 10.1111/aas.13070 . 29349777

[31] Kho ME, Molloy AJ, Clarke FJ, Reid JC, Herridge MS, Karachi T, et al. Multicentre pilot randomised clinical trial of early in-bed cycle ergometry with ventilated patients. BMJ Open Respir Res. 2019;6(1):e000383. Epub 20190218. doi: 10.1136/bmjresp-2018-000383 ; PubMed Central PMCID: PMC6424272. 30956804 PMC6424272

[32] Parry SM, Berney S, Warrillow S, El-Ansary D, Bryant AL, Hart N, et al. Functional electrical stimulation with cycling in the critically ill: a pilot case-matched control study. J Crit Care. 2014;29(4):695 e1–7. Epub 20140326. doi: 10.1016/j.jcrc.2014.03.017 . 24768534

[33] Kho ME, Molloy AJ, Clarke FJ, Ajami D, McCaughan M, Obrovac K, et al. TryCYCLE: A Prospective Study of the Safety and Feasibility of Early In-Bed Cycling in Mechanically Ventilated Patients. PLoS One. 2016;11(12):e0167561. Epub 20161228. doi: 10.1371/journal.pone.0167561 ; PubMed Central PMCID: PMC5193383. 28030555 PMC5193383

[34] Eggmann S, Verra ML, Luder G, Takala J, Jakob SM. Effects of early, combined endurance and resistance training in mechanically ventilated, critically ill patients: A randomised controlled trial. PLoS One. 2018;13(11):e0207428. Epub 20181114. doi: 10.1371/journal.pone.0207428 ; PubMed Central PMCID: PMC6235392. 30427933 PMC6235392

[35] Nickels MR, Aitken LM, Barnett AG, Walsham J, King S, Gale NE, et al. Effect of in-bed cycling on acute muscle wasting in critically ill adults: A randomised clinical trial. J Crit Care. 2020;59:86–93. Epub 20200530. doi: 10.1016/j.jcrc.2020.05.008 . 32585438

[36] Medicine ACoS. ACSM’s guidelines for exercise testing and prescription: Lippincott Williams & Wilkins; 2013. 10.1249/JSR.0b013e31829a68cf 23851406

[37] Thompson PD, Arena R, Riebe D, Pescatello LS, American College of Sports M. ACSM’s new preparticipation health screening recommendations from ACSM’s guidelines for exercise testing and prescription, ninth edition. Curr Sports Med Rep. 2013;12(4):215–7. Epub 2013/07/16. doi: 10.1249/JSR.0b013e31829a68cf . 23851406

[38] Tanaka H. COMBO exercise training for JUMBO benefits. Hypertens Res. 2011;34(9):997–8. Epub 2011/07/22. doi: 10.1038/hr.2011.109 . 21775999

