# Supplementary material for: Feasibility and safety of in-bed cycling/stepping in critically ill patients: A study protocol for a pilot randomized controlled clinical trial
Source: PLoS One. 2024 May 10;19(5):e0301368. doi: 10.1371/journal.pone.0301368 (PMC11086822; doi:10.1371/journal.pone.0301368)
Supplement: S1 File — (PDF) [file pone.0301368.s003.pdf]

중환자에서 사이클/스텝퍼를 이용한 다중운동중재의 실행가능성과 안전성

Feasibility and safety of in-bed cycling/stepping in critically ill patients

Version No: 1.5

Principal Investigator Affiliation: Department of Rehabilitation Medicine, Seoul National University Hospital

Name of principal Investigator: Woo Hyung Lee

Research summary

(※다음 각 항에 대해 비전문가도 이해할 수 있는 평이한 언어로 간단명료하게 기술해주시기 바랍니다.)

Research title	(Korean) 중환자에서 사이클/스텝퍼를 이용한 다중운동중재의 실행 가능성과 안전성
	(English) Feasibility and safety of in-bed cycling/stepping in critically ill patients
Principal investigator	Woo Hyung Lee
Research fund	This work was supported by the Korea Medical Device Development Fund (KMDFRnD, NTIS 202013C18) grant funded by the Korea government (the Ministry of Science and ICT, the Ministry of Trade, Industry and Energy, the Ministry of Health & Welfare, the Ministry of Food and Drug Safety).

Research purpose	This study is an exploratory pilot clinical trial in which multimodal exercise intervention with in-bed cycling/stepping is additionally provided to critically ill patients in bed rest along with conventional rehabilitation, compared to when only conventional rehabilitation is provided. This study aims to confirm the feasibility and safety of in-bed cycling/stepping intervention.
Study design	Pilot clinical trial
Study period	IRB approval ~ Dec 31, 2024
Participants	Critically ill patients in intensive care unit
Number of study participants	24
Vulnerable research participants	Since this study is conducted on critically ill patients hospitalized for various diseases, participants with impaired consent can be enrolled in the study.
Research method	1. Participants: Critically ill patients in intensive care unit<br>2. Randomization: Presence or absence of mechanical ventilation<br>(1) Control group: conventional rehabilitation (time: 20 minutes)<br>(2) Intervention group: conventional rehabilitation (time: 20 minutes) + in-bed cycling/stepping (time: 20 minutes, number of times: 1-3 times)

	3. Exercise methods using cycle/stepper: Multimodal exercise intervention<br>(1) Determined according to graded conditions<br>i. Richmond agitation sedation scale, RASS<br>ii. Motor power<br>iii. Function<br>(2) Exercise mode<br>i. Passive mode<br>ii. Active assistive mode<br>iii. Active mode<br>iv. Resistance mode<br>(3) Exercise intensity control according to RPE (Rating of perceived exertion) and heart rate standards<br><br>4. Clinical evaluation items<br>(0) Baseline clinical information<br>- Basic information: age, sex, weight (kg), height (cm)<br>- Premorbid modified Barthel index (MBI)<br>- Premorbid Functional Ambulation Category (FAC)<br>- Acute physiology and chronic health evaluation (APHACHE) II score<br>- Cardiopulmonary resuscitation within 24 hours prior to admission to the intensive care unit<br>- Primary diagnosis at intensive care unit admission<br>- Days from admission to intensive care unit to first intervention<br>(1) baseline evaluation:<br>RASS, CAM-ICU, Sum of MRC score, FAC, Hand grip strength, DEMMI, FSS-ICU<br>(2) at ICU discharge:<br>RASS, CAM-ICU, Sum of MRC score, Hand grip strength, DEMMI, FSS-ICU, SPPB, MBI<br>(3) at hospital discharge:<br>A sum of MRC score, Hand grip strength, DEMMI, SPPB, MBI, FES, ABC, SF-36v2<br>(4) at 1 month after hospital discharge:<br>A sum of MRC score, Hand grip strength, DEMMI, SPPB, MBI, FES,

	ABC, SF-36v2<br>(5) at 3 months after hospital discharge:<br>FES, ABC, SF-36v2<br>(6) others<br>i. Days to initiate ambulation, FAC≥2<br>ii. Mortality-28 days<br>iii. Duration of Mechanical Ventilation<br>iv. Length of stay in ICU (days)<br>v. Length of stay in hospital (days)<br>vi. Number of events requiring suspension or delay of conventional rehabilitation or cycling/stepping intervention<br>vii. Pittsburgh Rehabilitation Participation Scale (PRPS)<br>viii. Presence of occupational therapy and its application (dose)<br>ix. Presence of respiratory rehabilitation and its application (dose)<br>x. Adverse event and its reason<br>xi. Adherence: Total number of sessions of conventional rehabilitation, number of completed sessions of conventional rehabilitation, total number of sessions and time of multi-exercise intervention, number and time of completed sessions of in-bed cycling/stepping intervention
Efficacy evaluation	The number and percentage of completed in-bed cycling/stepping sessions, the duration and percentage of in-bed cycling/stepping sessions, and the number of cessations of in-bed cycling/stepping sessions, the interval from ICU admission to the first session of in-bed cycling/stepping, the number and percentage of completed conventional rehabilitation sessions, the duration and percentage of conventional rehabilitation sessions, the number of cessations of conventional rehabilitation sessions, the number of adverse events, the confusion assessment method for the ICU (CAM-ICU), Richmond agitation-sedation scale (RASS), de Morton mobility index (DEMMI), FAC, functional status score for the ICU (FSS-ICU), short physical performance battery score (SPPB), falls efficacy scale (FES), activities-specific balance confidence scale (ABC), sum of Medical Research Council (MRC) score, handgrip strength, modified Barthel index, 36-

	item short-form survey (SF-36) version 2.0, days to initiate ambulation (FAC ≥2), mortality-28 days, duration of mechanical ventilation, length of stay in the ICU, length of stay in the hospital, Pittsburgh rehabilitation participation scale (PRPS), concomitant occupational therapy and its application dose, concomitant pulmonary rehabilitation, and its application dose.
safety evaluation	Total mortality rate, number of rehabilitation stoppages (in each case of conventional rehabilitation and cycling/stepping intervention), all other adverse events related to rehabilitation
Expected effect and expected result	It is expected that safely performing rehabilitation exercises while lying down for critically ill patients will promote recovery of physical functions and improve independent living abilities, and hospitals will be able to accelerate their return to daily life by accelerating the discharge period.

Research plan

1. Research title

Feasibility and safety of in-bed cycling/stepping in critically ill patients

2. Research facility and address

Facility 1: Department of Rehabilitation Medicine, Seoul National University Hospital

Address: 101 Daehak-ro, Jongno-gu, Seoul, Republic of Korea

3. Name and title of principal investigator and co-researcher

1) Principal investigator

Woo Hyung Lee, Clinical assistant professor, Department of Rehabilitation Medicine, Seoul National University Hospital

2) Co-researcher

Hyung-Ik Shin, Professor, Department of Rehabilitation Medicine, Seoul National University Hospital

Sung Eun Hyun, Medical professor, Department of Rehabilitation Medicine, Seoul National University Hospital

3) Researcher

Soohyun Wi, Research professor, Department of Rehabilitation Medicine, Seoul National University Hospital

Jung Hyun Kim, Research professor, Department of Rehabilitation Medicine, Seoul National University Hospital

Soo Won Yu, Researcher, Department of Rehabilitation Medicine, Seoul National University Hospital

Kwan-Sik Sung, Physiotherapist, Department of Rehabilitation Medicine, Seoul National University Hospital

4) Manager of medical devices for clinical trials

Soohyun Wi, Research professor, Department of Rehabilitation Medicine, Seoul National University Hospital

4. Research requesting Institution

1) Name and address of research requesting institution Not applicable

2) Name and title of monitor Not applicable

5. Name and address of research fund support institution

Name: Korea Medical Device Development Fund (KMDFRnD) grant funded by the Korea government (the Ministry of Science and ICT, the Ministry of Trade, Industry and Energy, the Ministry of Health & Welfare, the Ministry of Food and Drug Safety)

Address: 5th floor, Seoul Chamber of Commerce and Industry, 39, Sejong-daero, Jung-gu, Seoul

6. Anticipated research period

IRB approval ~ Dec 31, 2024

7. Research target disease

Critically ill patients

8. Background and Objectives of the Research

1) Background of the Research

1-1) The Necessity of Intensive Care Unit (ICU) Rehabilitation

When admitted to the intensive care unit, patients often experience functional deconditioning, known as ICU-acquired weakness, which leads to neurological and muscular impairments [1]. Approximately 80% of patients admitted to the ICU suffer from neuromyopathy, muscle weakness, and muscle atrophy, with up to 20% of muscle loss possible within one week [2, 3]. Current conventional ICU rehabilitation programs still have limitations in improving patients' functional mobility. Thus, there is a need to provide additional rehabilitation using rehabilitation exercise equipment to achieve more efficient and effective rehabilitation.

1-2) The Necessity of personalized ICU Rehabilitation - Improving Cardiopulmonary Function and Muscle Strength, Conditioning

Recently, the international trend in rehabilitation emphasizes providing personalized treatments that consider cardiopulmonary function, muscle strength, and overall physical condition. Particularly, for effective functional improvement in critically ill patients, it is essential to implement exercises with appropriate intensity and mode personalized to their muscle strength and cardiopulmonary capacity. In the past, the importance of aerobic exercise in ICU rehabilitation was not strongly emphasized; however, aerobic exercise has now been recognized as crucial for enhancing cardiopulmonary function in critically ill patients [4]. Throughout the entire process of ICU rehabilitation, providing cycle/stepper adjustability, passive/active mode control, and exercise intensity control can be immensely helpful in offering personalized exercises based on the patient's

condition. In particular, incorporating multi-modal exercises using cycle/stepper equipment has been shown to be far more effective in producing exercise benefits compared to single-mode exercises [5, 6] Thus, there is a need for multi-modal interventions in tailored ICU rehabilitation.

1-3) The Necessity of personalized ICU Rehabilitation - Heart Rate-based Exercise Monitoring

Most critically ill patients are in a state of low physical activity, and it is essential to adjust exercise intensity based on their cardiopulmonary function before engaging them in an exercise program. Exercising at excessively low intensity may not yield the desired exercise benefits, making it necessary to consider the ratings of perceived exertion and apply appropriate exercise intensity levels accordingly.

2) Research Hypothesis and Objectives

2-1) Research Objectives

This study is an exploratory pilot clinical trial in which multimodal exercise intervention with in-bed cycling/stepping is additionally provided to critically ill patients in bed rest along with conventional rehabilitation, compared to when only conventional rehabilitation is provided. This study aims to confirm the feasibility and safety of in-bed cycling/stepping intervention.

9. Investigational Medicinal Product and Medical Device Code Names (or Generic Names of Active Ingredients), Quantity of Raw Materials, Dosage Forms, etc. (including Control Drugs)

1) Information on the Type and Product of Medical Device Clinical Trials

1-1) Type of Medical Device Clinical Trial

This clinical trial is an investigator-initiated exploratory pilot study and is also intended to confirm feasibility.

1-2) Medical Device Classification for the Devices Used in this Clinical Trial

The device used in this clinical trial falls under the "Powered Orthopedic Exercise Equipment" (A67020.02, Class 2).

Subcategory	Sub-subcategory	Class	Product Group
Orthopedic	Powered Orthopedic	2	Medical

and<br>Functional<br>Recovery<br>Equipment<br>(A67000)	Exercise Equipment<br>(A67020.02)		Stimulating<br>Device

Table 1. Medical Device Classification

1-3) Medical Device Intended Use, Target Disease, and Indications

① Intended Use: The powered device used to reconstruct muscles and recover joint movements.

② Target Disease and Indications: The powered orthopedic exercise equipment is used for rehabilitation purposes, including muscle reconstruction and joint movement recovery, for bedridden patients who have lower limb muscle weakness, and individuals with disabilities.

10. Inclusion Criteria, Exclusion Criteria, Target Sample Size, and Rationale for Calculation

1) Inclusion Criteria

- Critically ill patients

① Age 45 years and older

② Admission to the intensive care unit within 72 hours

③ Expected need for intensive care unit treatment for at least 48 hours

④ Functional Ambulation Category (FAC) score of 2 or higher before ICU admission

2) Exclusion Criteria

① Neurologic disorders

i. Central nervous system: Acute stroke, Advanced dementia, Hypoxic ischemic encephalopathy, HIE, and primary neurological disorders causing gait abnormalities with acute onset.

ii. Peripheral nervous system: Multiple sclerosis, Amyotrophic lateral sclerosis, Myathenia gravis, Acute inflammatory demyelinating

polyneuropathy

② Acute Deep venous thrombosis, Pulmonary embolism
③ Pneumothorax
④ Presence of external fixation devices, superficial metallic implants on the lower limbs, amputation, or severe dermatological conditions (e.g., decubitus ulcer, eschar).
⑤ Expected discharge from the intensive care unit within 3 days after admission
⑥ Pregnancy
⑦ Inability to obtain informed consent (refusal, lack of family members, family disagreement)
⑧ Life expectancy of less than 6 months

3) Target Sample Size and Rationale for Calculation

- Target Sample Size: A total of 24 participants (12 in the conventional rehabilitation group and 12 in the in-bed cycling/stepping group).
- Rationale: This study is an exploratory pilot clinical trial aiming to confirm the feasibility and safety of the cycling/stepping exercise intervention. Assuming a 20% loss for death and a 15% loss for follow-up, the required sample size was 12 per arm to be recruited in this study.

4) Recruitment Plan for Research Participants

Clinicians in the ICU at our hospital will collaborate to enroll patients for this clinical trial. Additionally, posters about this study are planned to be affixed on the noticeboard in front of the ICU, facilitating easy access to information regarding the clinical trial for representatives of patients. The principal investigator will provide a detailed explanation of the study to the participants' legal representatives since most admitted patients are critically or severely ill. The study will not exclude patients who are likely to participate based on their socioeconomic status. Every effort will be made to allow eligible patients to participate in the research, and patients will be informed of the purpose of the study to represent the entire population of critically ill patients receiving treatment at this institution.

11. Research Method

1) Specific Research Methods

① Conventional ICU Rehabilitation Protocol

Modified ICU Rainbow Mobilization Scales								
Mental Status		Unconscious		Conscious				
Rainbow mobilization Scale		Red	Orange	Yellow	Green	Blue	Dark blue	Violet
RASS		±3, ±4	±3, -4	±2 ≥	±2 ≥	±2 ≥	±2 ≥	±2 ≥
Motor power				grossly 1	grossly 2	UE ≤ 3, LE < 3	LE ≤ 3	LE ≥ 3
Check list		Safety Criteria: V/S, airway, bleeding, pain, lines						
Rehab program								
No activity		O						
ROM	Passive exercise		O	O	O	O	O	O
	Active assistive exercise			O	O	O		
	Active exercise				O	O	O	O
Sitting	Leaning or Tilting (in bed)		O	O				
	Supported sitting			O	O			
	long sitting/tailor sitting				O	O		
	Sitting on edge of bed					O	O	O
Standing	Sit to stand/Standing						O	O
	Marching on the spot						O	O
Ambulation	assisted gait & endurance training							O
Goal		Lying without contractures	Turning self	Sittng balance	Sit at edge	Standing & Transfer (bed-chair)	Assisted Gait	Gait endurance

i. Currently applied protocol at Seoul National University Hospital's intensive care unit.

ii. Progresses step-by-step based on the patient's consciousness, motor power, and functional status.

iii. The steps are categorized as Red → Orange → Yellow → Green → Blue → Dark blue → Violet, indicating improvement in the patient's consciousness, motor power, and functional status.

iv. Internationally, countries like the United States and Europe follow step-by-step rehabilitation protocols for critically ill patients [7, 8].

v. Conventional rehabilitation will be applied according to the patient's condition based on the steps in this study.

vi. This study aims to provide additional graded multimodal exercise intervention in addition to the existing conventional ICU rehabilitation protocol.

② In-bed cycling/stepping (Graded multimodal exercise) intervention

i. The in-bed cycling/stepping exercise intervention will be applied only to the intervention group (however, the conventional rehabilitation will be applied to both the control and intervention groups).

ii. Application period of multimodal exercise intervention: During the patient's stay in the intensive care unit (from ICU admission to ICU discharge).

iii. Patient position during in-bed cycling/stepping application: The patient will exercise comfortably, lying flat on the bed (supine position) or with the bed inclined at 30-40° (semi-Fowler's position).

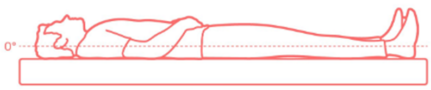

Supine position

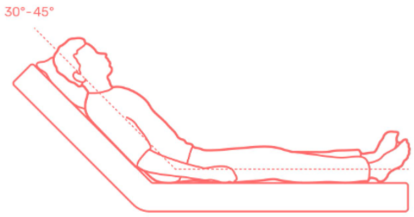

Semi-Fowler's position

iv. Application of cycling/stepping exercise: The patient's feet will be placed on the pedals of the device, and the forefoot and ankle will be secured with straps to strengthen the lower limb muscles through the in-bed cycling/stepping exercise.

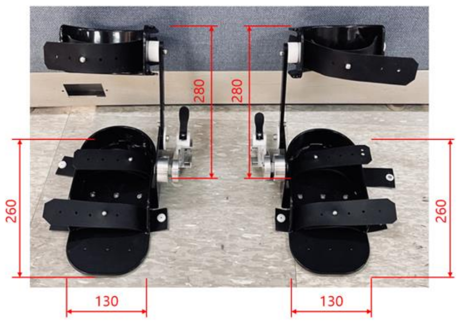

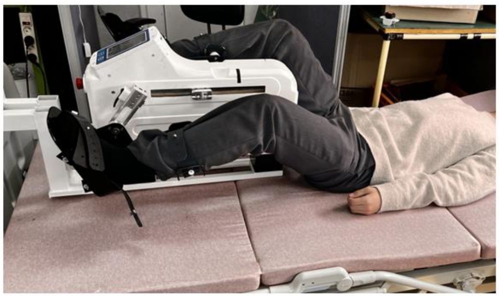

v. Types of Exercise during in-bed cycling/stepping application

A. The exercise options include passive, active assistive, active, and resistance exercise.

B. Passive Exercise: In this mode, the user performs the exercise passively according to the set rotation speed (rpm or revolutions per minute).

C. Active Assistive Exercise: When the user applies force above the set rotation speed (rpm or revolutions per minute) during passive exercise, the mode transitions to active exercise, where the user engages in the exercise actively. If the rotation speed drops below the set value, it reverts to passive exercise mode.

D. Active Exercise: This mode involves active exercise, and when the user applies force above the set rotation speed (rpm or revolutions per minute), it switches to active exercise mode, where the user performs the exercise actively.

E. Resistance Exercise: In this mode, the exercise load is set, and the patient moves the device on the patient's own to strengthen the muscles through resistance exercise.

F. In the Orange phase, in-bed cycling/stepping will not be applied.

G. In the Yellow phase, passive and active assistive exercise will be performed for 10 minutes each, sequentially, in one session.

H. In the Green phase, active assistive and active exercises will be performed for 10 minutes each, sequentially, in one session.

I. In the Blue phase, active assistive exercise and active exercise will be performed for 10 minutes each, sequentially, in one session.

J. In the Dark blue phase, active and strength exercises will be performed for 10 minutes each, sequentially, in one session.

K. In the Violet phase, active and strength exercises will be performed for 10 minutes each, sequentially, in one session.

vi. Number of in-bed cycling/stepping Interventions

A. The number of multi-exercise interventions will gradually increase based on the critical care rehabilitation protocol and the patient's condition.

B. From 1 to 2 days after admission to the intensive care unit, in-bed cycling/stepping will be applied once. From the following 1 to 2 days,

cycling/stepping will be applied twice. Subsequently, cycling/stepping will be applied a maximum of three times.

C. The duration of each in-bed cycling/stepping application will be 20 minutes, totaling 60 minutes for three in-bed cycling/stepping applications.

D. If there are no significant issues related to safety criteria [Refer to section 12 for anticipated side effects], the number of interventions will be increased. However, if significant problems occur, the number of interventions will be reduced.

vii. Exercise modes based on the number of in-bed cycling/stepping interventions (in-bed cycling/stepping usage ratio)

A. For one cycling/stepping intervention, the cycle or stepper will be used for 20 minutes.

B. For two cycling/stepping interventions, the cycle will be used for 20 minutes, and an additional 20 minutes will be added for the stepper.

C. For three cycling/stepping interventions, the cycle will be used for 20 minutes, the stepper for 20 minutes, and an additional 20 minutes will be added for either the cycle or stepper.

D. According to the conventional rehabilitation protocol and the patient's condition, exercise modes will gradually progress from passive to active assistive, active, and resistance (strength) modes.

E. During the Yellow stage of the conventional rehabilitation protocol, for one cycling/stepping intervention, the cycle or stepper will be used for 20 minutes for passive and active assistive exercises. Considering the patient's condition,

active assistive exercises will be applied as much as possible.

F. During the Dark blue and Violet stages of the conventional rehabilitation protocol, when the patient can perform active exercises, the resistance (strength) exercise mode will be gradually increased based on the patient's tolerance level [9].

③ Overview of Medical Devices Used in the Clinical Trial

i. Specification and Performance of the in-bed cycling/stepping Equipment

1. Main Body (Unit: mm): Mobile Cart + in-bed cyling/stepping Device

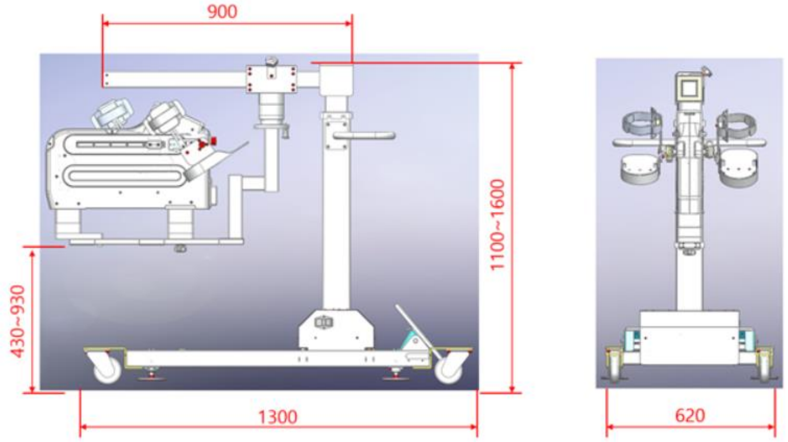

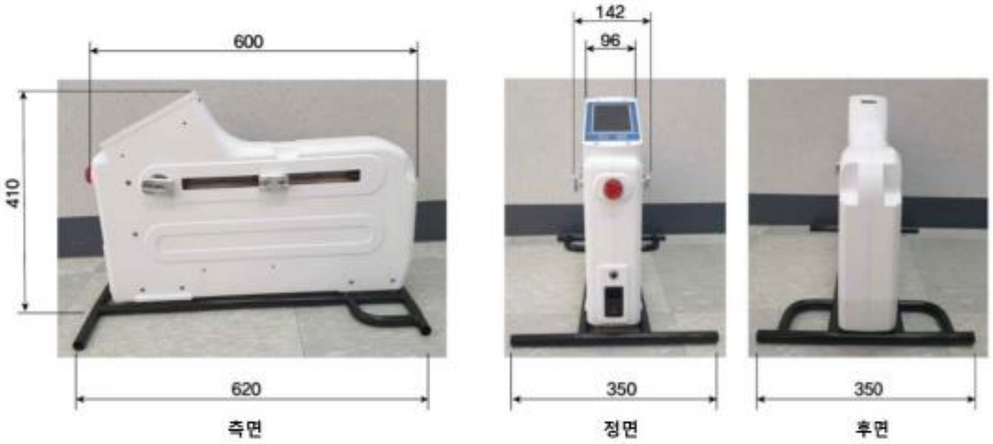

- Components (Unit: mm): Footrest, Emergency Switch, Power Cord

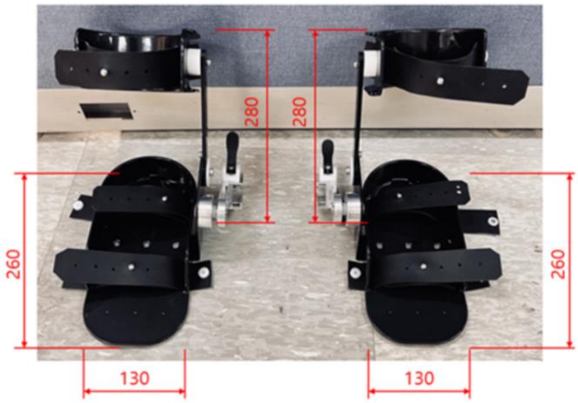

(1) 비상스위치

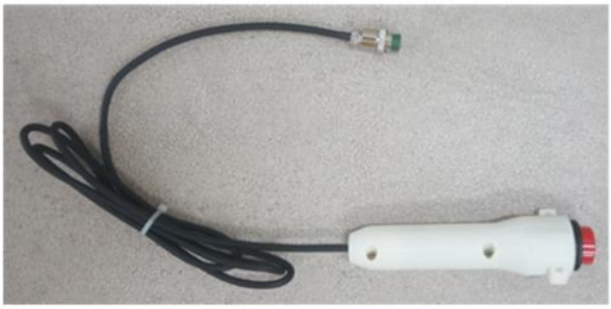

(2) 전원코드

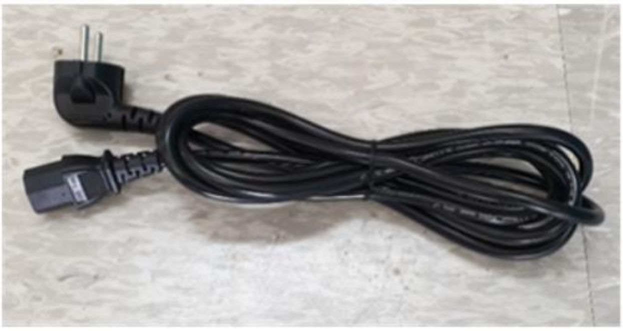

- Exterior Description

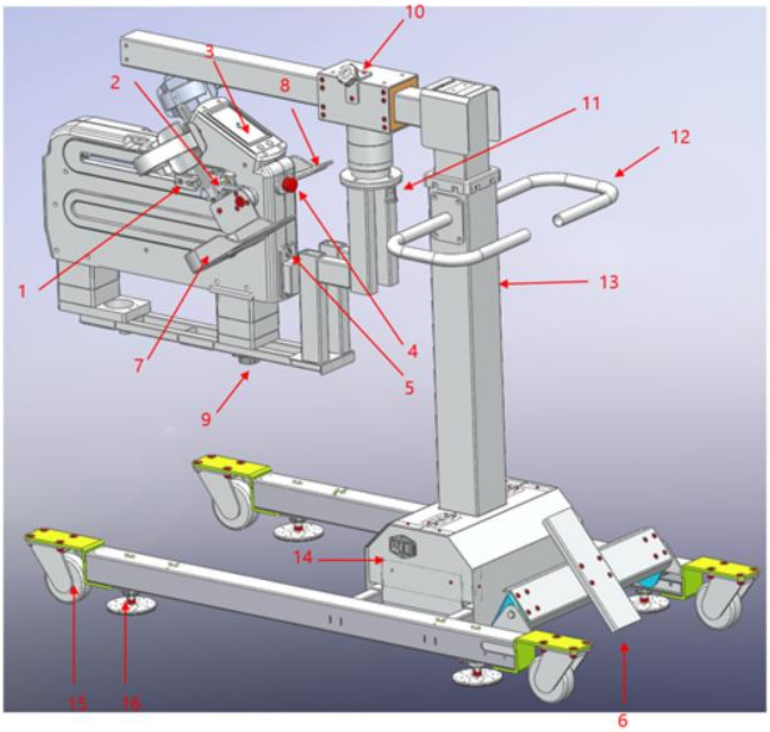

01. 스텝퍼운동용 발판 고정구
02. 사이클운동용 발판 고정구
03. 터치 스크린
04. 비상스위치
05. 비상스위치 연결 커넥터
06. 고정발 페달
07. 오른발 발판
08. 왼발 발판
09. 길이 조절 노브
10. 길이 조절 노브
11. 회전 노브
12. 이동 손잡이
13. 높이 조절부
14. 전원 케이블 커넥터
15. 이동 바퀴
16. 고정발

번호	명 칭	기 능
1	스텝퍼 운동용 발판 고정구	스텝퍼 운동시 발판을 고정
2	사이클운동용 발판 고정구	사이클 운동시 발판을 고정
3	터치 스크린	각종 값을 설정
4	비상스위치	비상상황에서 긴급 정지
5	비상스위치 연결 커넥터	외부 비상스위치 연결 커넥터
6	고정발 패달	고정발을 세팅하는 패달
7	오른발 발판	환자의 오른발 고정
8	왼발 발판	환자의 왼발 고정
9	길이 조절 노브	제품을 길이 방향으로 위치 고정
10	길이 조절 노브	제품을 길이 방향으로 길이 고정
11	회전 노브	제품의 회전 방향 고정
12	이동 손잡이	제품 이동하는 손잡이
13	높이 조절부	제품의 높이를 조절
14	전원 케이블 커넥터	전원 케이블 연결 커넥터
15	이동 바퀴	제품의 이동 바퀴
16	고정발	제품이 움직이지 않도록 위치 고정

2. Product Weight: The combined weight of the mobile cart and the cycling/stepper device is 77kg.

3. Product Performance

   3-1. User Mode

   A. Patient Information Storage

   B. Exercise Information Storage

   3-2. Exercise

A. Types of Exercise
   - Cycling Exercise: rotational movements
   - Stepper Exercise: linear and vertical movements
B. Exercise Modes
   - Passive exercise
   - Active assistive exercise
   - Active exercise
   - Strength (Resistance) Exercise (Exercise load options: 1-10 Watts)
C. Exercise Duration: 1~120 minutes
D. Exercise Speed
   - Cycling: 1~60rpm
   - Stepping: 5~20 cycles/minute
   - Exercise Load: 1-10 Watt
   - Metronome Setting: 0~220bpm (For example, 60bpm means one beat per second, with the sound playing once per second)
E. Safety equipment (Error Messages)
   - The device stops in case of any malfunction to protect the patient.
   - Emergency Stop, Speed Deviation from Set Parameters, Communication Errors, etc.

④ Duration of application of in-bed cycling/stepping Intervention: From when the patient no longer meets the criteria for [in-bed cycling/stepping Intervention and Delay or Cessation of ICU Rehabilitation] to ICU discharge.

⑤ Exercise Dose
  i. Conventional rehabilitation: Once a day, 20 minutes
  ii. In-bed cycling/stepping Intervention: 1-3 times a day, 20-60 minutes

⑥ Exercise Providers
  i. Conventional Rehabilitation - Hospital-affiliated Physical Therapist
  ii. In-bed cycling/stepping Intervention –Researcher Physical Therapist or Occupational Therapist

⑦ The protocol for conventional rehabilitation with in-bed cycling/stepping intervention has been developed by modifying the existing Seoul National University Hospital's critical care rehabilitation protocol (Rainbow Mobilization Scales).

Modified ICU Rainbow Mobilization Scales								
Mental Status		Unconscious		Conscious				
Rainbow mobilization Scale		Red	Orange	Yellow	Green	Blue	Dark blue	Violet
RASS		±3, ±4	±3, -4	±2 ≥	±2 ≥	±2 ≥	±2 ≥	±2 ≥
Motor power				grossly 1	grossly 2	UE ≤ 3, LE < 3	LE ≤ 3	LE ≥ 3
Check list		Safety Criteria: V/S, airway, bleeding, pain, lines						
Rehab program								
No activity		O						
ROM	Passive exercise		O	O	O	O	O	O
	Active assistive exercise			O	O	O		
	Active exercise				O	O	O	O
Sitting	Leaning or Tilting (in bed)		O	O				
	Supported sitting			O	O			
	long sitting/tailor sitting				O	O		
	Sitting on edge of bed					O	O	O
Standing	Sit to stand/Standing						O	O
	Marching on the spot						O	O
Ambulation	assisted gait & endurance training							O
Goal		Lying without contractures	Turning self	Sittng balance	Sit at edge	Standing & Transfer (bed-chair)	Assisted Gait	Gait endurance
Multimodal Exercise								
Cycle ergometer	Passive exercise			O				
	Active assistive exercise			O	O	O		
	Active exercise				O	O	O	O
	Resistive exercise						O	O
Stepper	Passive exercise			O				
	Active assistive exercise			O	O	O		
	Active exercise				O	O	O	O
	Resistive exercise						O	O

Figure 1. ICU Rehabilitation Protocol with in-bed cycling/stepping Intervention

⑧ Adjustment of Exercise Intensity and Heart Rate Monitoring Based on RPE Criteria

i. RPE(rating of perceived exertion): modified Borg scale

Rating	Descriptor
0	Rest
1	Very, Very Easy
2	Easy
3	Moderate
4	Somewhat Hard
5	Hard
6	*
7	Very Hard
8	*
9	*
10	Maximal

ii. Target RPE: The exercise intensity is adjusted to achieve a Rating of Perceived Exertion (RPE) level between 3 and 5.

iii. Heart Rate Monitoring During Exercise

1. Maximum Heart Rate: 220-Age [10]

2. Target Heart Rate: (220-Age) X (Maximum Heart Rate 70-80%)

⑨ Intervention Delay and Cessation Criteria [11, 12]: If any of the following conditions persist for more than 2 minutes during treatment initiation or intervention, conventional rehabilitation, and in-bed cycling/stepping intervention should be delayed or ceased, and if necessary, only passive

exercises will be provided.

i. Respiratory conditions
    1. Percutaneous oxygen saturation, SpO_2 < 88%
    2. Respiratory rate, RR > 40bpm
    3. Unsecure airway

ii. Cardiovascular conditions
    1. Mean arterial pressure < 60 mmHg or > 120 mmHg.
    2. Systolic blood pressure < 90 mmHg or > 200 mmHg.
    3. Heart rate < 50 bpm or > 140 bpm
    4. Arrhythmias causing hemodynamic instability.
    5. Concern for new myocardial ischemia.

iii. Neurologic conditions
    1. RASS scale ≥ 3
    2. Uncontrolled seizures

iv. Other Conditions
    1. Active bleeding, such as gastrointestinal bleeding
    2. Overt bleeding
    3. Body temperature ≥ 39℃
    4. Use of neuromuscular blockage within 4 hours.
    5. Severe pain, discomfort, or severe vomiting
    6. Ventilator asynchrony/extubation or detachment of artificial airway
    7. Open wounds.
    8. Patient fall during intervention.
    9. Patient requests to discontinue exercise

v. Catheterization Status of Femoral Artery or Vein (Applicable to cycling/stepping intervention)
    1. Presence of extracorporeal membrane oxygenation or Intra-aortic balloon pump
    2. Insertion of a perm catheter in the femoral vein

2) Control Group Setting and Randomization Method

① Control Group: Conventional rehabilitation based on the existing ICU rehabilitation protocol (for 20 minutes)

② Intervention Group: Conventional rehabilitation based on the existing ICU rehabilitation protocol (for 20 minutes) + in-bed cycling/stepping intervention

(for 20 minutes, 1-3 times).

③ Stratified randomization:

i. Criteria for Stratified Randomization

A. Presence or absence of mechanical ventilation.

ii. Randomization Method

A. The randomization table will be generated using a stratified block randomization method with a 1:1 allocation ratio to the treatment or control group and allocated through a web-based system.

B. The Medical Research Collaborating Center will create the randomization table at Seoul National University Hospital, and web-based randomization will be conducted.

3) Administration and Dosage of Examination Drug, Method of Administration and Use, Concomitant Therapy, and Reasons for Choosing Placebo when used as a Control

① Duration of Medical Device Usage

A. From admission to the intensive care unit (ICU) and initiation of intervention in the ICU to the time of discharge from the ICU.

② Usage of Medical Devices

A. Frequency of cycling/stepping Intervention [13]

i. According to the ICU rehabilitation protocol, The in-bed cycling/stepping Intervention frequency will gradually increase based on the patient's condition.

ii. After admission to the ICU and initiation of ICU rehabilitation, the usage will gradually increase.

iii. In the first 1-2 days after starting the in-bed cycling/stepping Intervention, the cycling/stepping will be applied once a day. If no significant problems occur, in the following 1-2 days, the in-bed cycling/stepping will be applied twice a day and then, if possible, up to a maximum of three times a day.

iv. The cycling/stepping application duration per session will be 20 minutes. If applied three times a day, 60 minutes will be required per day.

v. If no major issues corresponding to the criteria for delay or cessation of in-bed cycling/stepping intervention and ICU rehabilitation occur, the frequency will be increased. However, in case of significant problems, the frequency will be reduced under the supervision of the clinician.

③ Exercise Modes for in-bed cycling/stepping Intervention

i. Once the in-bed cycling/stepping intervention is applied, the cycle or stepper will be used for 20 minutes.

ii. When in-bed cycling/stepping intervention is applied twice, the cycle will be used for 20 minutes, followed by an additional 20 minutes on the stepper.

iii. When in-bed cycling/stepping intervention is applied three times, it will consist of 20 minutes of cycling, followed by 20 minutes on the stepper, and an additional 20 minutes of cycling or stepping

iv. The exercise modes will be progressed gradually based on the patient's condition, following the ICU rehabilitation protocol. These include passive, active-assistive, active, and muscle strength (resistance) exercise modes.

v. During the Yellow stage of the ICU rehabilitation protocol, when applying cycling/stepping intervention once, 10 minutes of passive exercise and 10 minutes of active-assistive exercise will be performed, considering the patient's condition and applying active-assistive exercise as much as possible.

④ Method of Medical Device Usage

i. Installation and operation of the medical device, as well as the use of the controller, should be referred to in the medical device's user manual (pages 6-17)

ii. Specific details of the in-bed cycling/stepping intervention protocol can be found in the detailed research methodology (pages 8-15)

4) Observation Items, Clinical Examination Items, and Observation Examination Methods

① Evaluation Items

A. Baseline clinical characteristics [8, 14-17]

i. Basic information:

- Age
- Gender: male/female
- Body weight (kg)
- Height (cm)
- Premorbid MBI (Modified Barthel index)
- Premorbid FAC, Functional ambulation category

- APACHE II score
- Whether cardiopulmonary resuscitation was performed within 24 hours before admission to the intensive care unit (ICU)

ii. Primary diagnostic category of ICU Admission
1. Cardiovascular disease
   - Myocardial infarction
   - Congestive heart failure
   - Rhythm disturbance
   - Cardiogenic shock
   - Peripheral vascular disease
   - Others
2. Respiratory disease
   - Acute respiratory distress syndrome
   - Exacerbation of chronic pulmonary disease
   - Others
3. Neurologic disease
4. Gastrointestinal disease
   - Acute liver failure
   - Gastrointestinal perforation
   - Others
5. Metabolic and renal disease
   - Acute renal failure
   - Others
6. Septic shock
7. Hemorrhagic shock
8. Malignancy
9. Burns
10. Trauma
11. Drug overdose
12. Other diseases
13. Unknown

iii. Comorbidity
1. Cardiovascular disease
   - Hypertension
   - Coronary heart disease

- Congestive heart failure
- Atrial fibrillation or Flutter
- Others

2. Chronic pulmonary disease
   - Asthma
   - Chronic obstructive pulmonary disease
   - Interstital lung disease
   - Others
3. Chronic liver disease
   - Cirrhosis
   - Chronic hepatitis B
   - Chronic hepatitis C
   - Others
4. Kidney disease
   - Dialysis
   - Others
5. Cancer
6. Immunosupression
   - Chemotherapy or radiotherapy within the past 6 months
   - Maglignant hemopathy
   - Neurotropenia
   - Transplant recipient
   - Treatment of corticosteroids
   - Others
7. Neurologic disease
   - Cerebrovascular disease
   - Dementia
   - Others

iv. Geriatric conditions
1. Dementia or cognitive impairment
2. Clinical frailty scale [18]
3. Past History of Falls within the last 3 months

v. Types of Treatment Received Before Study Registration
1. Surgery within 2 days of ICU admission
2. Emergency surgery between ICU admission and study registration

3. Noninvasive ventilation
4. Invasive mechanical ventilation
5. Continuous intravenous sedation
6. Continuous intravenous muscle relaxant
7. Continuous vasopressors
8. Renal replacement therapy

vi. Days from ICU Admission to First Intervention

B. Outcome

i. Feasibility and safety

- The number and percentage of completed in-bed cycling/stepping sessions, the duration and percentage of in-bed cycling/stepping sessions, and the number of cessations of in-bed cycling/stepping sessions, the interval from ICU admission to the first session of in-bed cycling/stepping, the number and percentage of completed conventional rehabilitation sessions, the duration and percentage of conventional rehabilitation sessions, the number of cessations of conventional rehabilitation sessions, the number of adverse events

ii. Mobility Assessment

- de Morton Mobility Index (DEMMI) score at the ICU discharge

iii. Level of consciousness Assessment

- CAM-ICU (Confusion Assessment Method for the ICU)
- RASS (Richmond Agitation-Sedation Scale)

iv. Mobility Assessment

- FAC (Functional Ambulatory Category)
- FSS-ICU (Functional Status Score)
- SPPB (Short Physical Performance Battery score)
- FES (Falls Efficacy Scale,)
- ABC (Activities-Specific Balance Confidence Scale)

v. Motor power Assessment

- Sum of MRC (Medical Research Council score)
- Hand grip strength

vi. ADL (Activities of daily living) Assessment

- MBI (Modified Barthel Index)

vii. QOL (Quaility of life) Assessment

- SF-36(36-Item Short Form Survey) version 2.0

viii. Others
- Days to initiate ambulation, FAC≥2
- Mortality-28 days
- Duration of Mechanical Ventilation
- Length of stay in ICU (days)
- Length of stay in hospital (days)
- Pittsburgh Rehabilitation Participation Scale (PRPS)
- Occupational Therapy Dosage
- Respiratory Rehabilitation Dosage

② Evaluation schedules

A. Screening
  i. Premorbid Mobility Assessment: FAC

B. Baseline
  i. Level of Consciousness Assessment: CAM-ICU, RASS
  ii. Mobility Assessment: FAC, DEMMI, FSS-ICU
  iii. Motor Power Assessment: Sum of MRC score, Hand grip strength

C. ICU Discharge
  i. Level of Consciousness Assessment: CAM-ICU, RASS
  ii. Motor Power Assessment: Sum of MRC score, Hand grip strength
  iii. Mobility Assessment: FAC, DEMMI, FSS-ICU, SPPB
  iv. Activities of Daily Living (ADL) Assessment: MBI

D. Hospital Discharge
  i. Motor Power Assessment: Sum of MRC score, Hand grip strength
  ii. Mobility Assessment: FAC, DEMMI, SPPB, FES, ABC
  iii. Activities of Daily Living (ADL) Assessment: MBI
  iv. Quality of Life (QOL) Assessment: SF-36v2

E. 1 Month After Hospital Discharge (Visit)
  i. Motor Power Assessment: Sum of MRC score, Hand grip strength
  ii. Mobility Assessment: FAC, DEMMI, SPPB, FES, ABC
  iii. Activities of Daily Living (ADL) Assessment: MBI
  iv. Quality of Life (QOL) Assessment: SF-36v2

F. 3 Months After Hospital Discharge (Self-report)
  i. Mobility Assessment: FES, ABC
  ii. Quality of Life (QOL) Assessment: SF-36v2

G. Daily

i. Others: days to initiate ambulation (FAC≥2) from the start of treatment, 28-day mortality rate, duration of mechanical ventilation, length of stay in ICU, length of stay in the hospital, Pittsburgh Rehabilitation Participation Scale (PRPS), Occupational therapy dosage, respiratory rehabilitation dosage, the number and percentage of completed in-bed cycling/stepping sessions, the duration and percentage of in-bed cycling/stepping sessions, and the number of cessations of in-bed cycling/stepping sessions, the interval from ICU admission to the first session of in-bed cycling/stepping, the number and percentage of completed conventional rehabilitation sessions, the duration and percentage of conventional rehabilitation sessions, the number of cessations of conventional rehabilitation sessions, the number of adverse events

③ Implementation of Evaluations

A. Evaluators: Hospital-affiliated physical therapists

B. Blinding Method: This is a single-blind, assessor-blinded clinical trial. The group allocation will be performed by an independent researcher who is not involved in evaluating outcomes and will be concealed to physiotherapists who are engaged in assessing outcomes. The participants and in-bed cycling/stepping intervention provider will be instructed not to disclose the allocation to the evaluator.

C. Unblinding:

i. After the end of the clinical trial, blinding will be maintained until the input of evaluation data is completed.

ii. Any unblinding prior to clinical trials due to accidental or severe adverse events should be documented.

		Study period							
Location		Admission-ICU				ICU-General ward		Hospital Discharge (D/C)	
Time point		Screening	Enrollment	Allocation	Baseline	ICU D/C	Hospital D/C	1 month after hospital D/C	3 months after hospital D/C
Enrollment	Eligibility screening	X							
	Informed consent		X						
	Randomization			X					
Assessment	FAC	X			X				
	RASS				X	X			
	CAM-ICU				X	X			
	Sum of MRC score				X	X	X	X	
	Hand grip strength				X	X	X	X	
	DEMMI				X	X	X	X	
	FSS-ICU				X	X			
	SPPB					X	X	X	
	MBI					X	X	X	
	SF-36v2						X	X	X
	FES						X	X	X
	ABC						X	X	X
	PRPS				←	—	→		
	Days to initiate ambulation (FAC≥2)				←	—	—	—	→
Safety	Adverse event				←	—	→		
Others	Mortality				←	—	→		
	Duration of mechanical ventilation (days)				←	→			
	Length of stay in ICU (days)				←	→			
	Length of hospital stay (days)				←	—	→		
	Occupational therapy and its application dose				←	—	→		
	Pulmonary rehabilitation and its application dose				←	—	→		
	The number of adverse events				←	—	→		
	The number of deferrals of each conventional rehabilitation and in-bed cycling/stepping				←	—	→		
	The number of total rehabilitation sessions				←	—	→		
	The number of completed rehabilitation sessions				←	—	→		

Figure 2. Evaluation items according to assessment time points during the study period

5) Distinctions from Existing Treatments and Research

① Additional in-bed cycling/stepping Intervention to Conventional rehabilitation protocol

i. In addition to conventional rehabilitation, in-bed cycling/stepping intervention is applied to improve functional mobility and mobilization. This includes lower limb strengthening and aerobic exercises using the cycle and stepper.

1. Cycling is known to have benefits as both a passive exercise for preventing lower limb contracture and an active exercise for aerobic training, increasing lower limb blood circulation, enhancing cardiopulmonary function, and strengthening lower limb muscles. Similarly, the stepping shows comparable effects, but the activated muscles and the degree of activation differ, potentially leading to more effective improvement in daily life functions, such as walking ability.
2. By applying both cycling and stepping simultaneously to critically ill patients, these exercises are expected to synergistically produce more beneficial effects than applying each individually.
3. The unique aspect of the medical device is that it allows both cycle and stepper to be applied together, facilitating efficient implementation of various exercises.

ii. Clear exercise delay or cessation criteria are defined to ensure safer exercise performance.

② Customized in-bed cycling/stepping Intervention for Study Participants

i. Based on the ICU rehabilitation protocol, stepwise application of passive exercises, active assistive exercises, active exercises, and muscle strength (resistance) exercises using cycle and stepper is tailored to the consciousness level, muscle strength, and functional status of critically ill patients.

ii. It is expected that providing various modes of exercise in combination with a stepwise application using cycle and stepper will be more effective [6].

③ Exercise Intensity Set by Rating of Perceived Exertion (RPE) with Heart Rate Monitoring

i. The exercise intensity is set at 3-5 levels based on the patient's RPE and is adjusted during exercise based on real-time heart rate monitoring to guide the exercise.

6) Benefits and Risks for Study Participants

① Expected Benefits for Study Participants

Existing research has reported that ICU rehabilitation is effective in reducing mechanical ventilation and improving muscle strength, endurance, and quality of life [19-21]. Additionally, the cycling/stepping intervention is expected to strengthen muscles and further enhance functional mobility.

② Expected Risks for Study Participants

The expected risks for study participants participating in this clinical trial are as follows:

- fall, fracture, severe pain, endotracheal tube removal, arterial line removal, central line removal, Adverse events that occurred during exercise included hemodynamic instability such as arrhythmia, bradycardia, systolic blood pressure > 200mmHg, and systolic blood pressure < 90mmHg, as well as desaturation ≥ 88%, Other minor adverse reactions observed during exercise were dyspnea, dizziness, tachypnea, or sinus tachycardia.
- Previous studies referenced in this research reported the occurrence of the following types and frequencies of adverse events [8].
  - the number of cases where exercise was discontinued:
    - Mean arterial pressure < 65mmHg: Control group (1.1%), Intervention group (0.1%)
    - Systolic blood pressure > 180mmHg: Control group (0.1%),

Intervention group (0.0%)

- Heart rate > 135 bpm: Control group (0.6%), Intervention group (0.1%)
- Heart rate < 45 bpm: Control group (0.1%), Intervention group (0.0%)
- Acute arrhythmia: Control group (0.4%), Intervention group (0.1%)
- Myocardial ischemia: Control group (-), Intervention group (-)
- Increase in respiratory rate of 36 bpm or more than 50%: Control group (0.8%), Intervention group (0.1%)
- Decrease in SpO2: Control group (1.0%), Intervention group (0.1%)
- Unplanned extubation: Control group (-), Intervention group (0.0%)
- Muscle fatigue: Control group (0.7%), Intervention group (0.5%)
- Pain: Control group (0.8%), Intervention group (0.6%)
- Agitation: Control group (1.0%), Intervention group (0.6%)
- Acute respiratory distress: Control group (1.1%), Intervention group (0.4%)
- Adverse reactions during conventional rehabilitation: Control group (1.0%), Intervention group (0.6%)
- Adverse reactions during lower limb cycling: Control group (-), Intervention group (3.5%)
- Total adverse reactions: Control group (1.0%), Intervention group (0.6%)
- Clinically significant adverse reactions requiring therapeutic intervention: Control group (1.0%), Intervention group (0.2%)
- Severe adverse reactions: Control group (-), Intervention group (-)

	Number of adverse events and interrupted sessions (% of sessions)		Number of patients experiencing at least one adverse event (%)	
**Adverse events leading to temporary interruption and/or postponing of mobilization session**	Usual care group 1190 sessions	Intervention Group 4159 sessions	Usual care group 154 patients	Intervention Group 158 patients
Occurrence of mean arterial pressure < 65 mmHg<br>*Including episodes leading to vasopressor dosage increase above 0.5μg kg min*	13 (1.1%)<br>9 (0.8%)	4 (0.1%)<br>4 (0.1%)	9 (5.8%)<br>6 (3.9%)	4 (2.5%)<br>3 (1.9%)
Occurrence of systolic arterial pressure >180 mmHg	1 (0.1%)	2 (0.0%)	1 (0.6%)	2 (1.3%)
Occurrence of heart rate >135 b./min	7 (0.6%	5 (0.1%)	6 (3.9%)	4 (2.5%)
Occurrence of heart rate <45 b./min	1 (0.1%)	1 (0.0%)	1 (0.6%)	1 (0.6%)
Occurrence of acute arrhythmia<br>*Including persistent arrhythmia with need of drug therapy*	5 (0.4%)<br>2 (0.2%)	4 (0.1%)<br>2 (0.0%)	3 (1.9%)<br>2 (1.3%)	4 (2.5%)<br>2 (1;3%)
Myocardial ischemia	0	0	0	0
Increase in respiratory rate above 36 b./min or by 50%	10 (0.8%)	3 (0.1%)	5 (3.2%)	2 (1.3%)
Drop in SpO2	12 (1.0%)	4 (0.1%)	10 (6.5%)	4 (2.5)
Unplanned extubation<br>*Including need of immediate reintubation*	0<br>0	1 (0.0%)<br>1 (0.0%)	0<br>0	1 (0.6%)<br>1 (0.6%)
Muscle fatigue	8 (0.7%)	21 (0.5%)	7 (4.5%)	17 (10.8%)
Pain	10 (0.8%)	23 (0.6%)	6 (3.9%)	8 (5.1%)
Agitation	12 (1.0%)	23 (0.6%)	8 (5.2%)	12 (7.6%)
Acute dyspnea and/or respiratory distress<br>*Including acute pulmonary edema with need of intensification of oxygen/ventilatory therapy beyond transient increase in oxygen flow in patients breathing spontaneously*	13 (1.1%)<br>1 (0.1%)	16 (0.4%)<br>1 (0.0%)	10 (6.5%)<br>1 (0.6%)	14 (8.9%)<br>1 (0.6%)
Adverse events that occurred during standardized mobilization program session	92/1190 (7.7%)	51/1387 (3.7%)	51 (33.1%)	31 (19.6%)
Adverse events that occurred during electrical muscle stimulation	-	8/1385 (0.6%)	-	2 (1.3%)
Adverse events that occurred during leg cycling exercises	-	47/1387 (3.5%)	-	30 (19.0%)
All adverse events	92/1190 (7.7%)	106/4159 (2.6%)	51 (33.1%)	50 (31.6%)
Clinically significant adverse events with need of therapeutic intervention	12/1190 (1.0%)	7/4159 (0.2%)	9 (5.8%)	7 (4.4%)
Serious Adverse Events	0	0	0	0

③ Compensation Policy

i. The study is covered by clinical trial insurance for compensation.

ii. Compensation Criteria

- Compensation will be provided for damages resulting from the multi-modal intervention conducted according to the clinical trial protocol.
- If a study participant experiences physical harm (including death) due to their participation in the clinical trial, compensation will be provided to the participant.
- In case of physical harm to a study participant due to their participation in the clinical trial, appropriate treatment will be provided through the responsible investigator before monetary compensation is confirmed.
- Damages incurred during the management of adverse events or during the process of addressing adverse reactions will also be considered for compensation.

iii. Exclusions from Compensation

- Cases, where physical harm (including death) is not directly related to

participation in the clinical trial, will be excluded from compensation.

- Incidents that are considered to have occurred regardless of the clinical trial, such as accidents or occurrences unrelated to the trial, will not be eligible for compensation.
- Participants who received only conventional rehabilitation as the control group and cannot be provided with therapeutic benefits will also be excluded from compensation.
- Damages resulting from the usual complications of the disease or disease progression will be excluded from compensation.
- If damages occur due to non-compliance with the instructions of the participant or their guardian, or non-adherence to the clinical trial protocol, intentional misconduct, or significant negligence, the compensation amount may be reduced, or the participant may be excluded from compensation.

7) Criteria for Discontinuation or Withdrawal

① Violation of inclusion/exclusion criteria.

② Withdrawal of consent by the participant or their guardian.

③ During the clinical trial, if it is deemed difficult to continue the trial due to a significant adverse event or abnormal clinical examination results.

④ Any other circumstance where the trial discontinuation is deemed beneficial to the study participant, as determined by the investigator.

8) Safety evaluation criteria, evaluation methods, and reporting methods, including side effects:

All adverse events (AEs) occurring during this clinical trial, including reported abnormal signs, symptoms, or diseases in research participants, are included in the safety evaluation. Vital signs measured during visits, clinical examinations, and adverse events reported by research participants are used to assess safety.

① Definition of adverse events:

i. "Adverse Event (AE)" refers to all harmful and unintended signs or symptoms or diseases that occur in research participants during the clinical trial, and it does not necessarily imply a causal relationship with the intervention in the trial.

ii. "Adverse Device Effect (ADE)" refers to all harmful and unintended reactions caused by the investigational medical device during the clinical trial, and it is presumed to have a causal relationship with the investigational medical

device.

iii. "Serious AE" refers to an adverse event or reaction that meets any of the following criteria:
  1. Results in death or poses a risk to life.
  2. Requires hospitalization or prolongs an existing hospitalization.
  3. Causes persistent or significant disability, long-term impairment, or functional loss.
  4. Causes a congenital anomaly or birth defect.

② Adverse event evaluation

i. Severity assessment
  1. Grade 1: Mild; the adverse event does not interfere with the research participant's normal activities (functioning) and causes minimal discomfort that the research participants can easily tolerate.
  2. Grade 2: Moderate; the adverse event causes some interference with the research participant's normal activities (functioning) and results in noticeable discomfort.
  3. Grade 3: Severe; the adverse event makes it impossible for the research participant to carry out normal activities (functioning).

ii. Causality assessment
  1. Definitely related
  2. Probably related
  3. Possibly related
  4. Probably not related
  5. Definitely not related
  6. Unknown

iii. Safety evaluation criteria
  1. In this clinical trial, adverse events are classified as any undesirable medical signs or symptoms occurring after the start of the trial that were not observed before the trial.
  2. Predicted side effects are also classified as adverse events, and the severity of adverse events is categorized as mild, moderate, or severe.
  3. Criteria, methods, and responses for the assessment of predicted side effects are provided for each item.

Predicted Safety Effects	Assessment Criteria	Assessment Method	Response
Fall	Fall from bed or	Confirmed by	Report immediately

	during mobility training resulting in injury	therapist and medical staff	to the attending medical staff and rehabilitation prescribing physician; additional tests, such as X-ray and CT, may be performed if necessary.
Fracture	Confirmation of bone fracture from an imaging test	Clinical Symptoms and X-ray Report	Report immediately to the attending medical staff and rehabilitation prescribing physician; additional tests, such as X-ray and CT, may be performed if necessary.
Severe pain	Severe pain strongly suspected by the critical care patient or with bruising or swelling in a specific area	occurring during or shortly after rehabilitation and lasting for more than three days	Confirm the pain in the affected area; report to medical staff and rehabilitation prescribing physician; additional tests, such as X-ray and CT, may be performed if necessary.
Endotracheal tube removal	Removal of endotracheal tube during rehabilitation	Confirmed by therapist and medical staff	Report immediately to medical staff, request ENT consultation for airway examination and additional tests if necessary, and inform the rehabilitation

			prescribing physician.
Arterial line removal	Removal of the arterial line during rehabilitation	Confirmed by therapist and medical staff	Report immediately to medical staff and inform the rehabilitation prescribing physician if necessary.
Central line removal	Removal of the central line during rehabilitation	Confirmed by therapist and medical staff	Report immediately to medical staff and inform the rehabilitation prescribing physician if necessary.
Hemodynamic Instability During Exercise	Arrhythmia, bradycardia, Systolic blood pressure > 200mmHg, Systolic blood pressure) < 90mmHg, SpO_2 ≤ 88% etc	Monitored through the intensive care unit (ICU) monitor by therapists and medical staff	Immediate reporting to medical personnel, conducting additional necessary tests, and informing the rehabilitation prescribing physician.
Other Mild Adverse Reactions	Dyspnea, dizziness, tachypnea or sinus tachycardia, agitation etc	Monitored by therapists and medical staff	Immediate reporting to medical personnel and conducting additional tests as necessary, and informing the rehabilitation prescribing physician.
Other Adverse Reactions	-	Monitored by therapists and medical staff	Immediate reporting to medical personnel and, conducting

			additional tests as necessary, and informing the rehabilitation prescribing physician.

③ Safety Evaluation Method

i. All observed predicted side effects and adverse reactions should be listed with detailed explanations.

ii. Record the frequency of adverse reactions associated with the investigational medical devices during the clinical trial and those unrelated to the investigational medical devices.

④ Adverse Reaction Reporting Method and Measures When Adverse Reactions Occur

i. Adverse Reaction Education:

The principal investigator shall provide education to the research personnel, research participants, or their representatives about all potential adverse reactions that may occur during the trial and educate them to report any events that occur after use.

ii. Serious Adverse Reaction/Medical Device Adverse Reaction Reporting Method:

- The principal investigator shall report all significant adverse reactions that occur during the research period to the IRB within 24 hours, regardless of the relationship with the investigational intervention.

Other significant adverse reactions considered by the researcher to be severe or indicating significant risks, contraindications, side effects, or precautions related to the investigational intervention shall be recorded as serious adverse reactions and reported to the IRB within 7 days from the date the researcher becomes aware of the event. Any other significant or unexpected adverse reactions shall be reported to the sponsor within 15 days from the date the researcher becomes aware of the event.

iii. Measures When Adverse Reactions Occur:

- During the clinical trial period, the principal investigator and research personnel must ensure the safety of the research participants, and in the event of an unexpected severe adverse reaction, take prompt and

appropriate measures to minimize the adverse reaction.

- If any predicted or unpredicted adverse reactions occur due to additional procedures or interventions beyond the standard clinical process during the clinical trial, the research personnel shall directly contact the investigator and provide guidance for applying for compensation, ensuring that the research participants receive the best possible medical treatment, and compensating the research participants when there is a reasonable causal relationship between the clinical trial and the injury.

iv. Tracking and Observing Adverse Reactions:

- The researcher must track and observe the research participants who experienced adverse reactions until their symptoms disappear and their condition stabilizes. If necessary, submit a report on the subsequent course of the adverse reactions to the IRB.

9) Data Analysis and Statistical Plan

The outcomes of the continuous variables will be analyzed using Mann-Whitney U test, whereas the discontinuous variables will be analyzed using the chi-square test or Fisher's exact test for variables between the two groups at the same time. Additionally, subgroup analysis will be conducted between the stratified groups Multiple imputation by chained equations algorithm will be used to handle missing data.

10) Data Management Plan

① Data Recording, Collection, Access, Protection, and Storage

i. When data are modified, the process of modification should be documented, and the original data should not be deleted. In case of changes or corrections in the contents of documented or electronically recorded case report forms, the modifications should be made in accordance with the written modification guidelines, ensuring that the original content can still be identified. The modification date, reason for modification, and the person making the modification should be recorded and signed.

ii. To ensure data management for the clinical trial, access to data by unauthorized individuals should be prevented by using individual IDs and passwords.

iii. A list of authorized personnel for data modification should be maintained.
iv. Copies of the data should be kept.
v. If data blinding (masking) is required for the specific clinical trial, the data entry and processing should be conducted while maintaining the blinding status.

11) Research Schedule

Months	1-3	4	5-6	7-32	33	34	35	36
FDA Medical Device Clinical Trial Plan Approval	●	●						
Medical Device Clinical Trial IRB Approval		●	●					
Medical Device Clinical Trial				●	●	●		
Statistical Analysis and Results Verification for Clinical Trial Data							●	●

12. Data and Safety Monitoring Plan

1) Monitoring Personnel

* **Monitoring Responsible Person:** Prof. Woo Hyung Lee, Clinical Assistant Professor, Department of Rehabilitation Medicine, Seoul National University Hospital

* **Monitoring Manager:** Research Prof. Soo Hyun Wee, Research Professor, Department of Rehabilitation Medicine, Seoul National University Hospital

2) Data and Safety Monitoring Items

***Study accruals items:** Clinical information from Case Report Forms (CRFs)

***Safety items:** [14, 16, 22]

① Fall
② Fracture
③ Severe pain
④ Endotracheal tube removal
⑤ Arterial line removal
⑥ Central line removal
⑦ Hemodynamic Instability During Exercise
    i. Arrhythmia

ii. Bradycardia
iii. Systolic blood pressure > 200mmHg
iv. Systolic blood pressure < 90mmHg
v. Desaturation ≥ 88%

⑧ Other Mild Adverse Reactions
i. Dyspnea
ii. Dizziness
iii. Tachypnea or Sinus tachycardia etc

3) Data and Safety Monitoring Methods and Frequency

① Data and Safety Monitoring Methods
During the participant's informed consent process, the research team explains the reporting system for safety monitoring. If any adverse reactions related to the research occur during the study, the entire research team will be immediately informed, and appropriate actions will be taken.

② Data and Safety Monitoring Frequency
Data and safety monitoring will be conducted after every 5 enrolled research participants.

4) Medical Device Adverse Reaction Reporting, Non-compliance with the Study, Reporting of Unexpected Problems

In the event of a serious adverse reaction, significant non-compliance with the study, or an unexpected problem occurring, the researcher must report it to the Institutional Review Board (IRB) through the monitoring responsible person within 15 working days from the date of awareness.

5) Criteria for Study Termination

- This study involves multimodal exercise intervention in critically ill patients during routine evaluation and treatment processes commonly performed in the clinical setting [8]. It is anticipated that the additional risks associated with the intervention will be low. Additionally, it is predicted that the likelihood of severe complications occurring, which would not have occurred if the intervention were not performed, is very low. However, if it is determined that the incidence rate of complications is significantly higher compared to before the study initiation, the study will be halted.
- If more than 25% of the intended research data is not acquired within 9 months

or if more than 50% is not acquired within 18 months, the research team will conduct a reevaluation through a meeting of involved parties and decide whether to resume the study.

13. Measures for Participant Safety and Protection

1) Basic Approaches to Ensure Research Ethics:

- The research team, including the principal investigator and personnel, will adhere to the Helsinki Declaration (2013 revision) and ICH-GCP guidelines and conduct the study after obtaining IRB approval. Before starting the study, all relevant information about the research, including the study objectives, potential effects, adverse reactions, and safety, will be sufficiently explained to the research participants and their legal representatives in a language they can understand. Voluntary informed consent will be obtained from the research participants and their legal representatives before their participation in the study.
- The purpose and methods of the study will be explained to potential research participants and their legal representatives through an informed consent form. Only those who understand the study's objectives and provide written consent will be eligible to participate. Records identifying the research participants will be kept confidential, and the identity of the research participants will remain confidential when publishing the study's results.

2) Informed Consent Process for Research Participants:

- For this study, the principal investigator (Prof. Woo Hyung Lee) or the research personnel will explain all relevant information about the research, including the study objectives, potential effects, adverse reactions, and safety, to potential research participants and their legal representatives at Seoul National University Hospital. The explanation will be provided in a language they can understand and will last for at least 10 minutes (if necessary, several days) to ensure sufficient understanding before obtaining voluntary informed consent for study participation.
- Minimizing the possibility of coercion or undue influence: Participation in the clinical trial will be completely voluntary, and adequate time (several days if needed) will be given to consider participation. The decision to participate will not be linked to the medical treatment provided at the institution for potential research participants.

3) Compensation Plan for Research Participants:

- There will be no remuneration for participation in the critical care rehabilitation and in-bed cycling/stepping exercise intervention conducted within the hospital.
- However, a case fee of 300,000 KRW will be provided for a follow-up visit one month after hospital discharge, and 50,000 KRW will be provided for a telephone follow-up three months after hospital discharge.
- The research responsibility includes not only covering all medical expenses for unforeseen complications but also providing compensation for any damages that may occur.

4) Measures for the Protection of Research Participants' Personal Information

- Patient medical record numbers and pathology numbers will be kept in a separate file under the responsibility of the principal investigator. The data will be coded so that individual identification is not possible through research data management. Alternatively, research data will be stored in password-protected files and kept in a locked facility with security measures. According to the medical device clinical trial management standards, research-related records will be retained for three years from the date of approval of the clinical trial results report. If the storage facility needs to retain certain documents related to personal information for more than three years for subsequent research, record-keeping, or accumulation purposes, such information will be managed to prevent individual identification. In cases where the trial results are published, the identity of the research participants will be kept confidential.

5) Additional Protective Measures for Vulnerable Research Participants

- If the research participants' capacity to provide informed consent is deemed difficult due to impaired decision-making abilities, their participation in the research will be obtained through their own assent and the consent of their legal representatives.
- The evaluation of consent capacity will consider factors such as whether the research participants can understand the information related to the study, handle the information logically, and express a clear choice regarding their desire to participate in the study.
- Since this study targets critically ill patients, most of whom have limited consent capacity, obtaining consent directly from the research participants is almost impossible. Therefore, consent will be obtained from their legal representatives, and the following protective measures will be strictly followed.

- Protective Measures for Adults with Impaired Consent Capacity (Refer to HRPP SOP ver.3.7)
  - This study targets critically ill patients, and most of them have impaired consent capacity.
  - It is anticipated that the expected benefits to the research participants will outweigh the foreseeable risks.
  - During the study, a third party, such as a research physical therapist or an ICU nurse, will always be present next to the critically ill patients to minimize potential adverse events and provide immediate assistance if needed.
  - Consent will be obtained from all research participants' legal representatives. The research team will directly explain the research and potential adverse events to the legal representatives and obtain their consent for the research participation on behalf of the research participants.
  - The evaluation of the research participants' consent capacity may include assessing their ability to understand the information related to the research, handle the information logically, and express a clear choice regarding their desire to participate. Since the ability to consent may fluctuate based on the research participants' condition, the understanding and willingness of the research participants for research participation will be repeatedly assessed.
  - Even if consent is initially obtained from the legal representatives, if the research participants gain the capacity to provide consent during the course of the study, the researchers must obtain consent from the research participants.
  - In emergency situations where obtaining prior consent from the research participants is not possible, consent from accompanying legal representatives must be obtained, and if there is no accompanying legal representative, written approval from the IRB must be obtained. In such cases, the principal investigator and researchers must inform the research participants or legal representatives about the research as soon as possible and obtain their continued consent for participation in the research.

14. Storage and Disposal Methods for Human-Derived Materials

Not applicable.

15. References

1. Dang SL. ABCDEs of ICU: Early mobility. Crit Care Nurs Q. 2013;36(2):163-8.
2. Field-Ridley A, Dharmar M, Steinhorn D, McDonald C, Marcin JP. ICU-Acquired Weakness Is Associated With Differences in Clinical Outcomes in Critically Ill Children. Pediatr Crit Care Med. 2016;17(1):53-7.
3. Fan E, Zanni JM, Dennison CR, Lepre SJ, Needham DM. Critical illness neuromyopathy and muscle weakness in patients in the intensive care unit. AACN Adv Crit Care. 2009;20(3):243-53.
4. Jang MH, Shin MJ, Shin YB. Pulmonary and Physical Rehabilitation in Critically Ill Patients. Acute Crit Care. 2019;34(1):1-13.
5. Ho SS, Dhaliwal SS, Hills AP, Pal S. The effect of 12 weeks of aerobic, resistance or combination exercise training on cardiovascular risk factors in the overweight and obese in a randomized trial. BMC Public Health. 2012;12:704.
6. Tanaka H. COMBO exercise training for JUMBO benefits. Hypertens Res. 2011;34(9):997-8.
7. Miranda Rocha AR, Martinez BP, Maldaner da Silva VZ, Forgiarini Junior LA. Early mobilization: Why, what for and how? Med Intensiva. 2017;41(7):429-36.
8. Fossat G, Baudin F, Courtes L, Bobet S, Dupont A, Bretagnol A, et al. Effect of In-Bed Leg Cycling and Electrical Stimulation of the Quadriceps on Global Muscle Strength in Critically Ill Adults: A Randomized Clinical Trial. JAMA. 2018;320(4):368-78.
9. Burtin C, Clerckx B, Robbeets C, Ferdinande P, Langer D, Troosters T, et al. Early exercise in critically ill patients enhances short-term functional recovery. Crit Care Med. 2009;37(9):2499-505.
10. Nes BM, Janszky I, Wisloff U, Stoylen A, Karlsen T. Age-predicted maximal heart rate in healthy subjects: The HUNT fitness study. Scand J Med Sci Sports. 2013;23(6):697-704.
11. Hodgson CL, Stiller K, Needham DM, Tipping CJ, Harrold M, Baldwin CE, et al. Expert consensus and recommendations on safety criteria for active mobilization of mechanically ventilated critically ill adults. Crit Care. 2014;18(6):658.
12. Berney S, Haines K, Skinner EH, Denehy L. Safety and feasibility of an exercise prescription approach to rehabilitation across the continuum of care for survivors of critical illness. Phys Ther. 2012;92(12):1524-35.
13. Ross AG, Morris PE. Safety and barriers to care. Crit Care Nurse. 2010;30(2):S11-3.
14. Schaller SJ, Anstey M, Blobner M, Edrich T, Grabitz SD, Gradwohl-Matis I, et al. Early, goal-directed mobilisation in the surgical intensive care unit: a randomised controlled trial. Lancet. 2016;388(10052):1377-88.
15. Kitzman DW, Whellan DJ, Duncan P, Pastva AM, Mentz RJ, Reeves GR, et al. Physical Rehabilitation for Older Patients Hospitalized for Heart Failure. N Engl J Med. 2021;385(3):203-16.
16. Schweickert WD, Pohlman MC, Pohlman AS, Nigos C, Pawlik AJ, Esbrook CL, et al. Early physical and occupational therapy in mechanically ventilated, critically ill patients: a randomised

controlled trial. Lancet. 2009;373(9678):1874-82.
17. Wunsch H, Angus DC, Harrison DA, Linde-Zwirble WT, Rowan KM. Comparison of medical admissions to intensive care units in the United States and United Kingdom. Am J Respir Crit Care Med. 2011;183(12):1666-73.
18. de la Cruz M, Bruera E. Approach to the older patient with cancer. BMC Med. 2013;11:218.
19. Lipshutz AK, Gropper MA. Acquired neuromuscular weakness and early mobilization in the intensive care unit. Anesthesiology. 2013;118(1):202-15.
20. Kress JP. Clinical trials of early mobilization of critically ill patients. Crit Care Med. 2009;37(10 Suppl):S442-7.
21. Engel HJ, Needham DM, Morris PE, Gropper MA. ICU early mobilization: from recommendation to implementation at three medical centers. Crit Care Med. 2013;41(9 Suppl 1):S69-80.
22. Bailey P, Thomsen GE, Spuhler VJ, Blair R, Jewkes J, Bezdjian L, et al. Early activity is feasible and safe in respiratory failure patients. Crit Care Med. 2007;35(1):139-45.
